# How Tropical Biodiversity Gets Multiplied: Documentation of Entomological Proofs from the Family Nepticulidae, Tiny Lepidopteran Leaf Miners **

**DOI:** 10.3390/insects16090964

**Published:** 2025-09-15

**Authors:** Jonas R. Stonis, Andrius Remeikis, Arūnas Diškus, Svetlana Orlovskytė

**Affiliations:** 1 Entomology Department, State Scientific Research Institute Nature Research Centre, Akademijos g. 2, LT-08412 Vilnius, Lithuania; remeikis.andrew@gmail.com (A.R.); s.orlovskyte@gmail.com (S.O.); 2 Education Academy, Vytautas Magnus University, K. Donelaičio g. 58, LT-44248 Kaunas, Lithuania; diskus.biotaxonomy@gmail.com

**Keywords:** new species, pygmy moths, *Serjania*-feeding, *Stigmella*, species diversity, species groups, taxonomy

## Abstract

The factors that generate the extraordinary biodiversity of tropical regions are likely multifaceted. In this study, we tested our working hypothesis that a mosaic of contrasting habitats plays a key role in shaping the diversity of tropical pygmy moths (Lepidoptera: Nepticulidae). To explore this, we selected four ecologically distinct localities in Honduras and investigated whether closely related but distinct Nepticulidae species occur in different habitats. Collected specimens were dissected, and their genital structures photographed and analyzed. Molecular divergence was assessed using DnaSP, with Maximum Likelihood phylogenetic trees constructed in MEGA. Bayesian phylogenetic analyses and mitotype networks construction (TCS algorithm) were also performed. Our findings are presented in two case studies. In Case Study 1, three closely related yet distinct species were discovered in separate localities; in Case Study 2, four other closely related species were found in environmentally different sites. Comparative material from Colombia and Peru was also examined. Based on integrated morphological and molecular analyses, eight new species were identified and formally described. We conclude that contrasting environmental conditions exert selective pressures, and that morphologically and genetically similar species—likely derived from a common ancestor—have diverged, exemplifying the process of adaptive radiation.

## Simple Summary

The mechanisms underlying the richness of tropical biodiversity have been widely studied, yet they remain the subject of ongoing debate. In this study, we explored the role of contrasting ecosystems in shaping species diversity, focusing on pygmy moths (Lepidoptera: Nepticulidae)—a family of tiny moths whose larvae are highly specialized leaf miners. We hypothesized that Nepticulidae diversity is strongly influenced by environmental variation across habitats and, consequently, by the diversity of host plants these habitats support. To test this hypothesis, we selected Honduras as a focal region—a relatively small tropical country characterized by strikingly diverse ecosystems. Prior to our research, virtually no studies on Nepticulidae had been conducted in Honduras, making it a “blank slate” for biodiversity exploration and an ideal setting for assessing the ecological drivers of species richness. We proposed that surveying ecologically distinct but geographically proximate localities would reveal different species assemblages, thereby supporting our hypothesis that habitat heterogeneity acts as a “hidden engine” driving tropical biodiversity. Our results confirmed this assumption: we describe eight new species of Nepticulidae, based on both morphological and molecular data, all found in habitats with varying environmental conditions despite their morphological similarities.

## 1. Introduction

Among the best-known patterns of life on Earth is the geographical trend of increasing biodiversity from the poles to the equator. Tropical biodiversity, in particular, is characterized by extraordinary richness. The factors that generate and sustain this biodiversity have been extensively studied; however, they remain subjects of debate, with no single explanation achieving full consensus [1,2,3,4,5,6]. Ecological hypotheses often attribute latitudinal diversity gradients to biological responses driven by abiotic conditions, especially those shaped by solar radiation. For instance, the niche hypothesis suggests that adaptations to specific combinations of abiotic factors and biotic interactions enable tropical species to achieve greater specialization, facilitating more precise resource partitioning among a larger number of species [7]. Another hypothesis emphasizes evolutionary dynamics, suggesting that the tropics support richer biodiversity due to greater effective evolutionary time, which refers to prolonged favorable conditions or uninterrupted speciation, as well as faster evolutionary processes [8]. Recent discussions have also highlighted the role of kinetics, specifically, temperature-dependent biological processes: higher tropical temperatures drive increased metabolic rates, ecological interactions, and coevolutionary processes, which together drive the generation and maintenance of greater biodiversity [5,7]. For a comprehensive overview of the hypotheses explaining tropical biodiversity, we particularly recommend the article “Why Are There So Many Species in the Tropics?” by James H. Brown [7]. We propose that these hypotheses are not mutually exclusive; rather, they offer complementary explanations that are applicable across different taxa and contexts. Together, various factors converge to establish the tropics as outstanding hotspots of life on Earth.

When studying pygmy moths (Lepidoptera: Nepticulidae), tiny moths whose larvae are specialized leaf miners of plants (for references, see [9,10]), we hypothesized that the species diversity of Nepticulidae is strongly influenced by ecosystems (habitats) that vary in environmental conditions and, consequently, by the variety of host plants they support. For our targeted study, we specifically selected Honduras, a relatively small tropical country where significantly contrasting ecosystems provide an ideal opportunity to explore how different environments contribute to species richness.

It is important to note that prior to our study, no research on Nepticulidae had been conducted in Honduras, except for two publications about our recent discoveries from the tropical dry forests along the Pacific coast [11,12] and the tropical humid forests along the Caribbean coast [12]. Thus, regarding the diversity of pygmy moths, Honduras was essentially a “blank slate” (or *tabula rasa*), and the combination of the absence of previous research and the country’s diverse habitats made it an ideal setting for our investigation.

**The Working Hypothesis.** In this study, we proposed that a survey of closely situated localities with differing environments could lead to the discovery of different species—providing evidence to support our hypothesis that a mosaic of habitats acts as a “hidden engine” that drives and multiplies tropical biodiversity.

**The Goal of the Study.** We aimed to find evidence supporting our working hypothesis by documenting Nepticulidae species that, although morphologically very similar, represent distinct taxa occurring in localities with different environmental conditions. For our study plots in Honduras, we selected four localities with contrasting environments (listed and illustrated in the Materials and Methods Section). Common species, characterized by their dark wings and distinct pale transverse fascia (which also makes their recognition easier), belonging to the most widespread and speciose Nepticulidae genus *Stigmella* Schrank, were selected as model taxa for our search for counterparts across different ecosystems and habitats. It is worth mentioning that in climatically relatively homogeneous environments, some Trans-Palaearctic *Stigmella* species may achieve distribution ranges extending up to approximately 8000–9000 km, as can be inferred from the findings of previous studies [13,14].

We also hope that the data presented in this paper will contribute to the general knowledge of these peculiar, tiny micromoths and stimulate further research in tropical regions and other often-neglected areas.

## 2. Materials and Methods

**Materials.** The materials for this paper were obtained by one of the authors’ team members. Since 2023, Prof. Dr. Jonas R. Stonis, Senior Researcher at the State Scientific Research Institute Nature Research Centre, has been visiting the Delegation of the European Union to Honduras and conducting voluntary research on the biological diversity of Honduran forests. During this mission, he sampled leaf-mining Lepidoptera. This initiative was part of two long-term programs between the European Union and Honduras: the Memorandum of Understanding between the Republic of Honduras and the European Union (“Forest Partnership”) and the Multiannual Indicative Program of the European Union for Honduras for 2021–2024, which includes Priority Area 1: “Sustainable Management of Natural Resources and Climate Change”, with the participation of the Honduran Institute of Forest Conservation, Protected Areas, and Wildlife (ICF). The material used in this paper will be deposited in the collection of the Museum für Naturkunde (MfN), Berlin, Germany, following publication.

Additionally, we examined materials from related species collected in other countries. These include the type-series specimens of *Stigmella foreroi* Stonis & Vargas, which were collected by us in Colombia under collecting permit No. 2019007511-1-000 issued by the Autoridad Nacional de Licencias Ambientales, Bogotá, Colombia, and deposited at the Pontificia Universidad Javeriana (Bogotá, Colombia). The specimens that formed the basis for the description of a new species, *S. serjanica* Stonis & Diškus, sp. nov., were collected by us in Peru, thanks to the Environmental Programme at the Andes Office of NGO Derecho Ambiente y Recursos Naturales, Peru, and training courses and fieldwork for the project “Rapid Assessment of Biodiversity Plots of Critical Value in the Provinces Chanchamayo and Satipo, Peru”, in cooperation with the Baltic-American Biotaxonomy Institute in 2017–2018.

**Figure 1:**
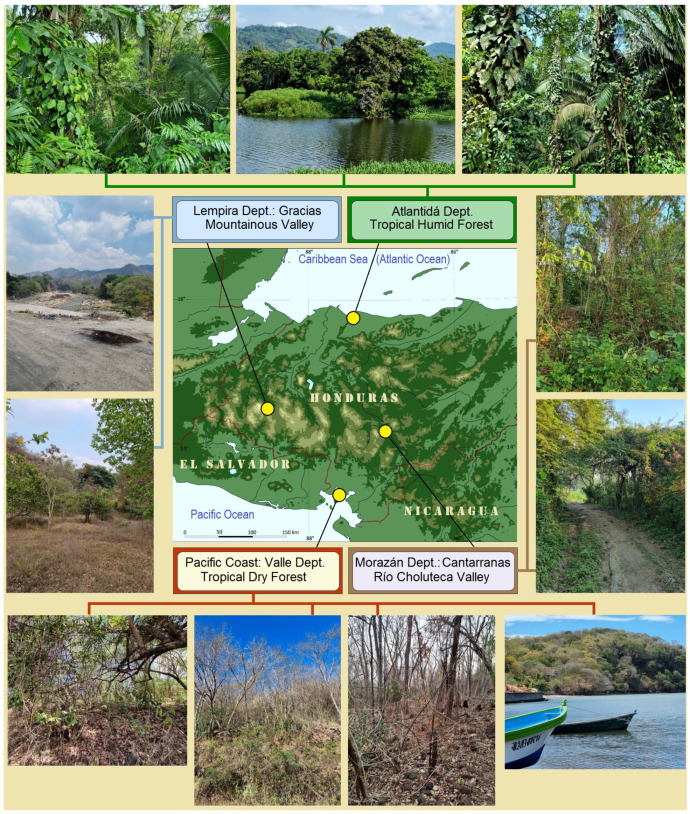
Study localities in Honduras specifically selected for research conducted during the dry season (2023–2025). Note: For the naming of tropical forests, we follow the most comprehensive recent terminology classification by the Hierarchy Revisions Working Group, U.S. Department of Agriculture, Forest Service [25,26].

**Study Localities.** In Honduras, four localities from four different departments were selected (Figure 1). These localities are situated 100–180 km apart, with the two most distant (on the Caribbean and Pacific coasts) separated by approximately 260–280 km. Despite their relative proximity, each site represents a distinct environment, with two showing strongly contrasting climatic conditions—the tropical dry forests and tropical humid forests.

The forests along the Pacific coast and in some interior mountain valleys of Honduras are referred to by slightly different names in the literature [15,16,17,18,19,20,21]; however, we consistently use the term”tropical dry forests”, both here and in our previous work (e.g., [11]). Likewise, although the tropical forests along the Caribbean coast are sometimes described as “tropical moist forests” [20,22,23,24], we follow the most comprehensive and recent classification [25,26] and refer to them as ”tropical humid forests”. Regardless of terminology, the climatic and biotic differences between the dry and humid forest types in Honduras are striking.

During this research, the environmental characteristics of the study plots were not analyzed in detail. For the brief descriptions provided here, we relied on our field observations and relevant literature. The ecological diversity of Honduras’ natural regions has been discussed to some extent in earlier works [24,27].

*Tela and La Ceiba*, located on the northern, Caribbean coast of Honduras in the Atlántida Department, lie within a region characterized by a tropical humid climate. The study plots were situated at elevations ranging from 10–30 m above sea level (Tela and its surrounding areas) to approximately 100 m (La Ceiba, right bank of the Río Cangrejal). The area experiences a warm, humid climate that contrasts with the cooler, higher-altitude regions of the country. The region has a distinct wet season from May to October and a much less pronounced dry season from November or December to April or early May—corresponding with our field research period.

This area forms part of the Mesoamerican Biological Corridor [24] and encompasses a variety of ecosystems, including lowland tropical humid forests (rainforests). It supports a remarkable diversity of flora and fauna, particularly species adapted to humid environments. The study sites ranged from nearly pristine habitats to areas influenced by human activities, such as plantations and urban development.

*Cantarranas* (officially also known as San Juan de Flores) is located in the Río Grande o Choluteca valley, on the northern foothills of La Tigra Mountain (2185 m) in Francisco Morazán Department, Honduras. The locality features a transitional climate between tropical humid and tropical dry. The study plots, situated at elevations of 600–760 m above sea level, experience a more moderate climate than the Pacific lowlands. This area has a distinct wet season from May to October, followed by a drier period from November to April (during which our research was conducted). Local ecosystems include a mix of tropical dry and moist forests, supporting biodiversity adapted to both altitudinal variation and fluctuating moisture levels. The region hosts a rich diversity of flora and fauna, including species adapted to seasonal rainfall and mid-elevation conditions. The nearby study plots were located in small, disturbed secondary forest remnants along a village path and river, embedded within heavily agricultural land dominated by extensive sugarcane plantations.

*The Pacific coast—San Lorenzo and the islands in the Gulf of Fonseca*, located in the Valle Department—lies within a region characterized by a tropical dry climate, sometimes unofficially referred to locally as”tierra caliente”. The study plots, situated at low elevations (10–30 m above sea level), experience a harsh dry season lasting five to six months. This prolonged dryness, combined with consistently high temperatures, starkly contrasts with the more humid climates found elsewhere in Honduras. This region supports distinctive tropical dry forests, shaped by extended drought and irregular rainfall. Despite the climatic challenges, the area hosts a surprisingly rich diversity of flora and fauna, all highly adapted to arid conditions. Study sites ranged from slightly disturbed natural habitats to areas heavily modified by agriculture and pastureland.

*Gracias*, located in the Lempira Department, lies in the western highlands of Honduras, nestled in a valley at the foothills of Cerro Las Minas (2870 m)—the highest mountain in the country. The study site is situated at approximately 800 m above sea level and experiences a subtropical highland climate, markedly different from the tropical lowlands. This region has a distinct wet season from May to October and a dry season from November to April (coinciding with our research period). Local ecosystems include both subtropical dry and moist forest zones, supporting a rich diversity of flora and fauna adapted to altitudinal and moisture gradients. Although the broader region is biologically diverse, our specific study plot was located in a heavily altered landscape dominated by pastures and orchards, and did not reflect the more pristine surrounding cloud forests or pine woodlands.

In addition to material from Honduras, related specimens were also obtained from Colombia and Peru for comparative analysis (Figure 2).

**Time and Duration.** All fieldwork in Honduras was conducted during the same seasonal window—February to April—in the years 2023, 2024, and 2025. This period corresponds to the dry season in the country, when adult Nepticulidae activity is likely at its peak. Additionally, the dry-season conditions allowed for more consistent and efficient use of light traps, since evening rain showers—so frequent during the wet season—were largely absent and therefore did not disrupt nocturnal collecting.

**Figure 3:**
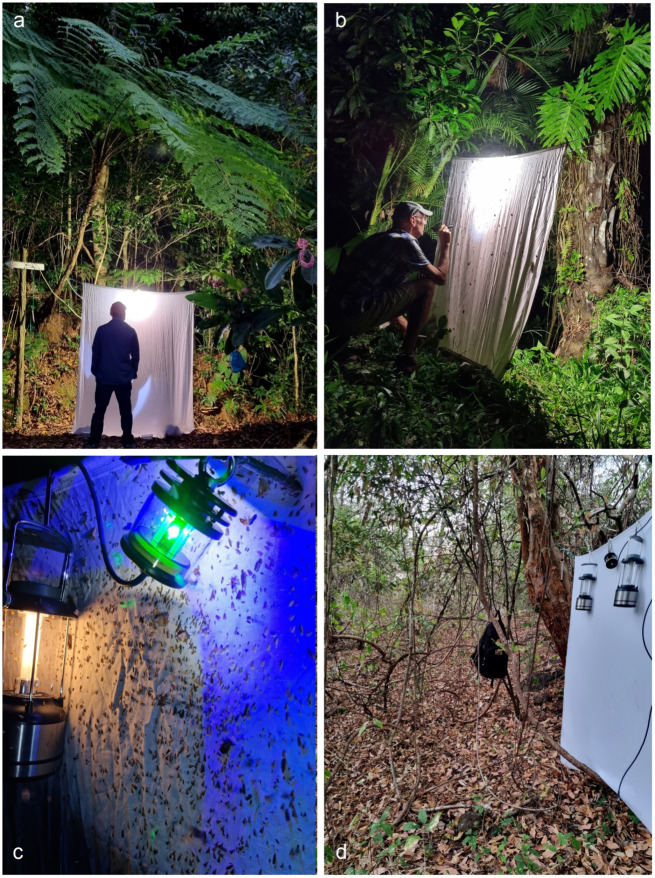
Nighttime sampling for Nepticulidae: (**a**,**b**) attracting moths with light from a Philips ML bulb (220–230 V, 160 W); (**c**,**d**) in locations without electric mains, sampling with fluorescent lanterns powered by D-cell batteries and a LepiLED lamp, specially designed for collecting nocturnal moths [28], operated with 5–13 V DC from power bank batteries.

**Collecting Methods.** During our fieldwork in Honduras, we used the same collection techniques and equipment at all study localities. Where access to mains electricity was available, moths were attracted using a Philips ML 160 W 220–230 V bulb suspended in front of a white screen (Figure 3a,b). In areas without electricity, we employed a modern LepiLED lamp and fluorescent lanterns powered by D-cell batteries [10] (Figure 3c,d). The LepiLED, specifically designed for collecting nocturnal moths [28], is lightweight, compact, and operates on 5–13 V DC supplied by power bank batteries. When mining larvae were available, we reared them to adults using the traditional method described and illustrated in the recent monograph on the Neotropical Nepticulidae [10].

**Specimen Dissection and Documentation.** The procedures for specimen dissection, species identification, and description adhered to the protocols outlined in previous studies [10,13,29,30]. Male genital capsules were carefully extracted following maceration of the abdomen in 10% KOH, followed by cleaning and mounting with the ventral side facing up. In many instances, the phallus was removed from the genital capsule and mounted under a separate cover slip on the microscope slide. Abdominal pelts were preserved in this study. Permanent preparations on microscope slides were photographed and analyzed using a Leica DM2500 microscope (Leica company, Wetzlar, Germany) with a Leica DFC420 digital camera. Adult specimens were measured and examined using a Lomo MBS-10 stereoscopic microscope (Lomo company, St. Petersburg, Russia), while images were taken using a Leica S6D stereoscopic microscope coupled with a Leica DFC290 (Leica company, Wetzlar, Germany) digital camera. For illumination of adult specimens, a stereomicroscope ring light LED 60 (Motic company, Xiamen, China) was employed, directly attached to the stereomicroscope lens, with adjustable intensity and a color temperature range of 7000 to 11,000 K, delivering 8000 Lux at a distance of 100 mm. Scanning electron microscopy (SEM) images were captured with a FEI Quanta 250 microscope (FEI Europe B.V. company, Eindhoven, The Netherlands) (State Scientific Research Institute Nature Research Centre, Vilnius). Leaf-mine photographs were taken with cameras that have the SuperMacro application, including a Canon Power Shot S-3 (Olympus corporation, Tokyo, Japan). Some leaf mines have been photographed in the wild using smartphones (especially those with good photography capabilities, such as a Samsung Galaxy S with a Leica lens (Samsung Electronics Co., Ltd., Seoul, South Korea).

**Molecular Analysis.** DNA extraction, amplification, and purification are described in our recent paper [11]. Sanger sequencing was performed at BaseClear B.V. (Leiden, The Netherlands). The successfully obtained 657-base-pair (bp) sequences of the cytochrome c oxidase subunit 1 (CO1-5′) were manually aligned using BioEdit v.7.2.5 [31] and deposited in the NCBI GenBank database [32] (accession IDs: PQ630806–PQ630816, PX230080–PX230082). For further analysis, sequences of related *Stigmella* and the outgroup were downloaded from the BOLD platform [33]. Molecular divergences were assessed with the DnaSP v.6 program [34]. The Maximum Likelihood (ML) trees (GTR + G + I model, 10,000 bootstrap replicates) were constructed using MEGA v.7.0 [35]. The ClustalX, software (version 2.0) [36] was used to convert Fasta format to Nexus. Bayesian phylogenetic analysis was performed (GTR + G + I model, 10 million generations) with the MrBayes v.3.2.3 program [37]. The Bayesian trees were processed with FigTree v.1.4.4 [38]. The mitotype networks were designed using the TCS Network algorithm [39], implemented in the PopArt v.1.7 program (http://popart.otago.ac.nz; accessed on 28 November 2024). Species delimitation was estimated using the ASAP [40] and bPTP [41] methods.

**Abbreviations for Institutions and Specimen Depositories.** BRG—Biosystematics Research Group, currently based at the State Scientific Research Institute Nature Research Centre (NRC), Vilnius, Lithuania; MfN—Museum für Naturkunde, formerly known as the Museum für Naturkunde der Humboldt-Universität zu Berlin or Museum für Naturkunde/Leibniz-Institut für Evolutions- und Biodiversitätsforschung, Berlin, Germany; NRC—State Scientific Research Institute Nature Research Centre, Vilnius, Lithuania; PUJ—Departamento de Biología, Pontificia Universidad Javeriana (Xavierian Pontifical University), Bogotá, Colombia.

## 3. Results

### 3.1. Case Study 1: Examination of Stigmella ruralis sp. nov. and Its Affiliates—Species with Morphological and Genetic Similarities

**Figure 4:**
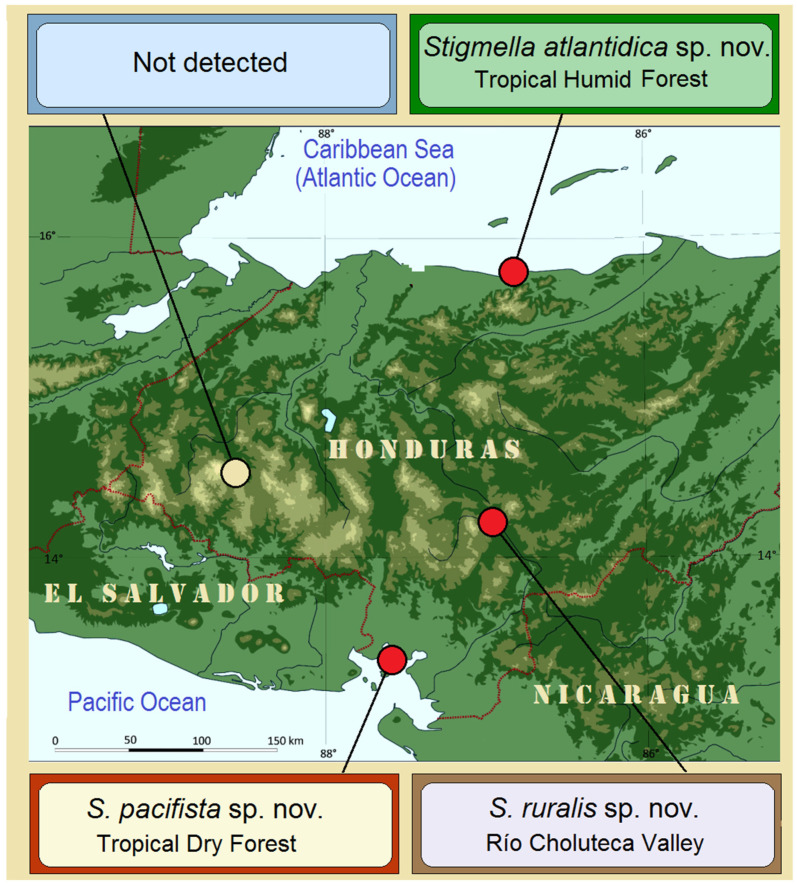
Discovery of *Stigmella ruralis* sp. nov. and its affiliates from different study localities in Honduras.

**Figure 5:**
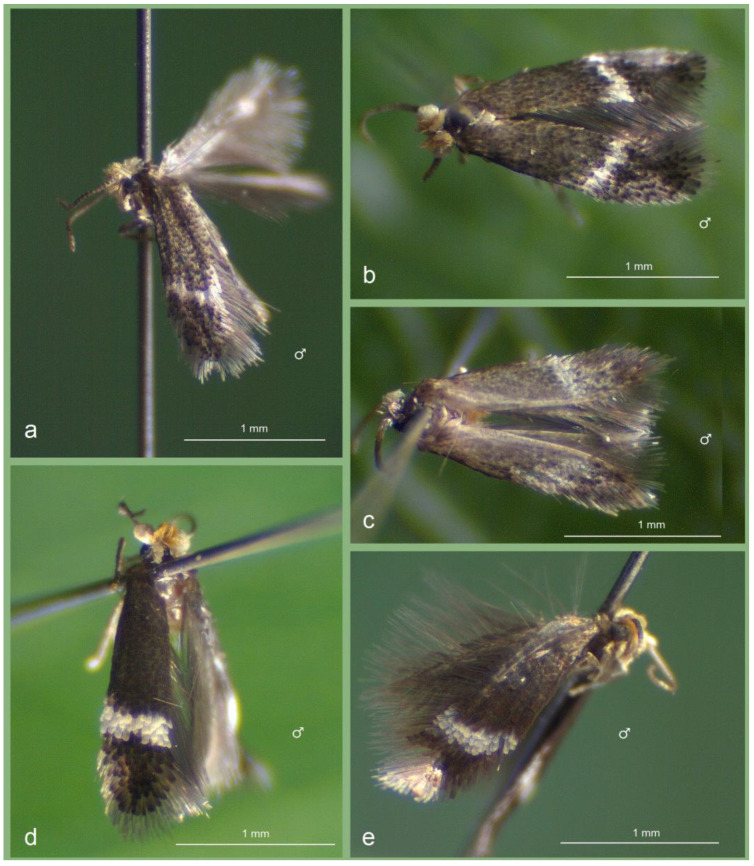
Adults of *Stigmella ruralis* sp. nov. and its related affiliates detected from different study localities in Honduras: (**a**,**b**) *S. ruralis* Stonis & Remeikis, sp. nov., from the Río Choluteca Valley; (**c**) *S. pacifista* Stonis & Remeikis, sp. nov., from the tropical dry forest of the Pacific coast of Honduras; (**d**,**e**) *S. atlantidica* Stonis & Diškus, sp. nov., from the tropical humid forest of the Caribbean (Atlantic) coast of Honduras.

During fieldwork in three of the four selected localities, three closely related species were discovered: *Stigmella ruralis* Stonis & Remeikis, sp. nov. from the Río Choluteca Valley; *S. pacifista* Stonis & Remeikis, sp. nov. from the tropical dry forest of the Pacific coast of Honduras; and *S. atlantidica* Stonis & Diškus, sp. nov. from the tropical humid forest of the Caribbean (Atlantic) coast of Honduras (Figure 4 and Figure 5). All these newly discovered species were determined to represent undescribed taxa and are described below.

#### 3.1.1. Description of *Stigmella ruralis* Stonis & Remeikis, sp. nov.

*ZooBank Registration.* https://zoobank.org/NomenclaturalActs/aa84690c-c6b4-4071-b841-1e6faa16602c (accessed on 27 August 2025).

*Material examined*. Holotype: ♂, HONDURAS, Francisco Morazán Department, Cantarranas (=San Juan de Flores), ca. 660–690 m, 14°15′50″ N, 87°01′35″ W–14°16′17″ N, 87°01′07″ W, 10–15 February 2024, leg. J.R. Stonis, genitalia slide no. RA1239♂ (MfN). Paratypes: 2 ♂, 1 ♀, the same label data as holotype, genitalia slide no. RA1211♂, RA1237♀ (MfN); 1 ♂, Cantarranas, 660 m, 14°15′57″ N, 87°01′31″ W, 18–23 April 2023, leg. J.R. Stonis, genitalia slide no. AD1199♂ (aberrant) (MfN).

*Diagnosis.* The new species differs from congeneric *Stigmella* species by the combination of the following characters: a distinctly bilobed uncus; two pointed apical processes of the valva; a unique U-shaped thickening between the valvae; slender, curved sublateral processes of the transtilla; a specific band of the phallus bearing tiny, triangular and slender spine-like cornuti; and an oblique white fascia on the forewing. This new species differs from the most similar and presumably closely related Caribbean species, *S. virginica* Remeikis & Stonis, 2017 (illustrated in [11]), by the following diagnostic characters: the dark forewing with a distinctive postmedian fascia (in *S. virginica*, the forewing is pale, sparsely irrorated with dark scales, and the fascia is subapical and weakly defined); the larger apical processes of the valva (in *S. virginica*, the apical processes are unusually small); and the sublaterally rounded vinculum (in *S. virginica*, the vinculum bears small, process-like sublateral extensions).

*Barcodes.* We barcoded two male paratype specimens from Cantarranas, Morazán Department, collected between 10 and 15 February 2024: one specimen with the genitalia slide no. RA1211 and the other undissected. Their sequences have been deposited in GenBank (accession IDs: PX230081, PX230082).

*Male* (Figure 5a,b). The forewing length ranges from 1.5 to 1.6 mm; the wingspan ranges from 3.5 to 3.6 mm (*n* = 3). The palpi and the frons are glossy cream. The frontal tuft is pale beige-orange. The collar and the scape are glossy cream. The antenna is significantly shorter than half the length of the forewing; the flagellum is grey-black on the upper side, greyish cream on the underside, with 23 segments. The thorax, the tegula, and the forewing are densely speckled with grey-black scales; the forewing fascia is slightly postmedian, oblique, and comprises glossy white scales. The fringe is glossy white apically, grey on the tornus; the fringe-line is weakly developed, formed of grey-black-tipped scales. The underside of the forewing is grey-black, without spots or androconia, with some purple iridescence. The hindwing and its fringe are grey, without androconia. The legs are black-grey dorsally, greyish cream or cream ventrally. The abdomen is grey-black to brownish black dorsally, glossy cream to brown ventrally; the tufts are rubbed or absent; the genital plates are covered with glossy white cream or pale brown scales.

*Female*. The forewing length is 1.6 mm; the wingspan is 3.6 mm (*n* = 1). The scape and the collar are yellowish cream. The antenna is covered with grey scales on the upper side. Otherwise, the female is similar to the male.

*Male genitalia* (Figure 6). The genital capsule ranges from 235 to 255 µm in length and from 120 to 140 µm in width. The uncus is split into two triangular lobes caudally, with a narrow gap between them. The gnathos has two slender caudal processes arising from a slender transverse plate. The valva bears two pointed apical processes; the inner lobe of the valva is almost straight. A unique U-shaped thickening is present between the valvae at the base. The transtilla bears slender, outwardly curved sublateral processes. The juxta is indistinct or membranous and may be absent. The vinculum is short, distally tapered, almost truncated, and lacks pronounced lateral lobes. The phallus measures 155 to 165 µm in length, with a width ranging from 40–45 µm in the median part to 55–60 µm at the apical and basal ends. The vesica bears a wide band of tiny triangular and slender spine-like cornuti, with the latter prevailing in the apical half; the cathrema is weakly developed and sometimes rather indistinct.

**Figure 7:**
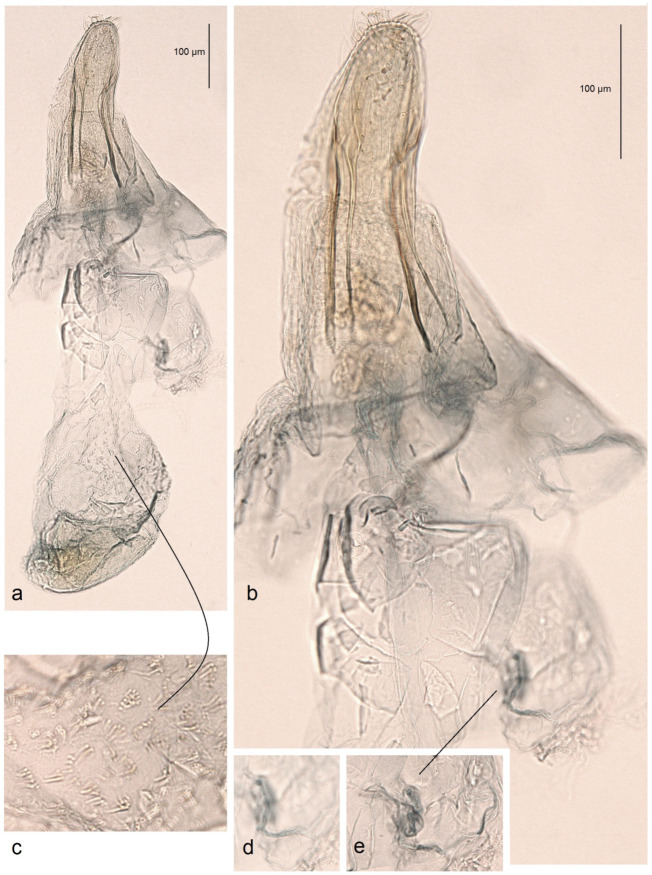
Female genitalia of *Stigmella ruralis* Stonis & Remeikis, sp. nov., from Cantarranas, Río Choluteca Valley, Honduras, genitalia slide RA1237, paratype (MfN): (**a**) general view; (**b**) enlarged view of the ovipositor; (**c**) comb-like pectinations; (**d**,**e**) details of the ductus spermathecae and a vesicle.

*Female genitalia* (Figure 7). The total length is approximately 900 µm. The ovipositor is relatively long and slender but rounded distally. The anterior and posterior apophyses are almost equal in length and very slender. The accessory sac is large but weakly chitinized. The ductus spermathecae is sinuous, without distinctive coils. The corpus bursae bears comb-like pectinations.

*Bionomics.* Adults fly from February to April. The specimens of the type series were attracted to light. Otherwise, the biology of the species remains unknown.

*Distribution*. This species is known from a single locality: Cantarranas (=San Juan de Flores), from narrow remnant strips of forest surrounded by heavily developed agricultural plantations in mountainous central Honduras, at an elevation of approx. 650 m.

*Etymology*. The species name *ruralis* is derived from the Latin adjective *ruralis*, meaning “of the countryside” or “rural”. Although *ruralis* is masculine in form, it is also used with feminine nouns in Latin, which aligns with its application here to the feminine genus *Stigmella*. This name was chosen to reflect the species’ discovery in a rural, agricultural area in Honduras.

#### 3.1.2. Description of *Stigmella pacifista* Stonis & Remeikis, sp. nov.

*ZooBank Registration*. https://zoobank.org/NomenclaturalActs/af37401d-db81-4076-8d49-6900e30a0f5b (accessed on 27 August 2025).

*Material examined*. Holotype: ♂, HONDURAS, Pacific coast, Isla Zacate Grande, El Moray (Restaurante Terra Mar), ca. 10 m, 13°21′33″ N, 87°35′57″ W, 18–19 March 2023, leg. J.R. Stonis, genitalia slide RA1238 (MfN).

**Figure 8:**
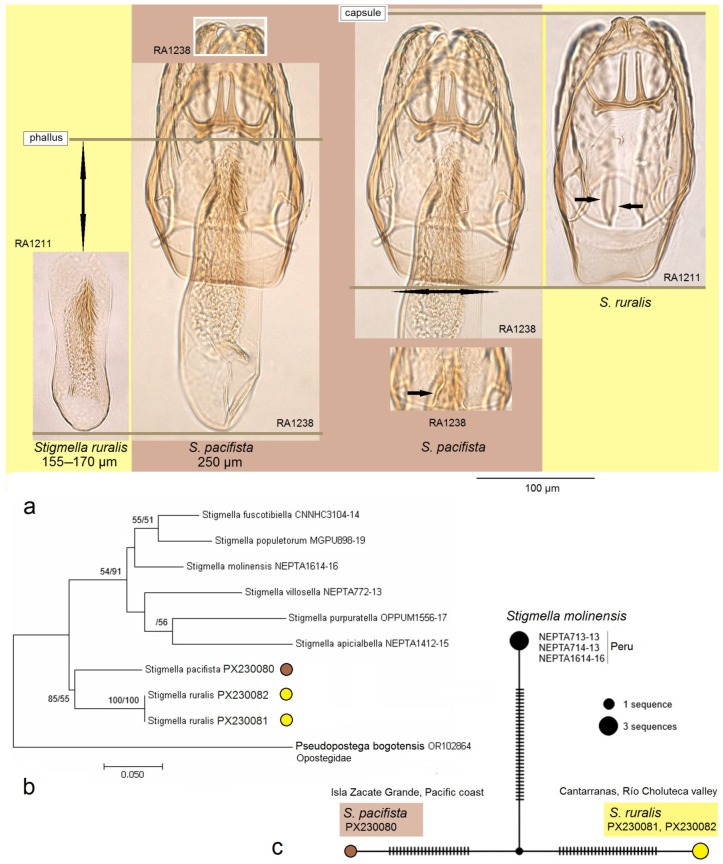
Comparison of *Stigmella pacifista* sp. nov. with *S. ruralis* sp. nov.: (**a**) the male genitalia; (**b**) phylogenetic relationships among selected *Stigmella* species based on 657 bp-long mtDNA CO1-5′ sequences and the GTR + G + I evolutionary model; topology reconstructed using Maximum Likelihood and Bayesian inference; branch statistics, separated by slashes: Maximum Likelihood probability (10,000 replicates)/Bayesian posterior probability (10,000,000 generations) in % (only values above 50 are displayed); (**c**) mitotype network of the 657 bp-long mtDNA CO1-5′ sequences of *S. pacifista* sp. nov., *S. ruralis* sp. nov., and the resembling species *S. molinensis* van Nieukerken & Snyers, constructed using the TCS Network algorithm; the size of each circle is proportional to the frequency of the mitotype; the smaller, unnamed circle represents the mitotype predicted to exist but not sampled in the study; the dashes on the connecting lines indicate hypothesized mutational steps.

*Diagnosis.* Externally and in the male genitalia, the new species closely resembles *S. ruralis* sp. nov. (described above); however, it differs from the latter by a significantly longer phallus, a wider vinculum, the absence of the U-shaped basal thickening between the left and right valvae (Figure 8a), and by molecular data (Figure 8b,c) (see also *Discussion* for further details).

*Barcode.* We barcoded the male holotype from Isla Zacate Grande, the Pacific coast of Honduras, with the genitalia slide label no. RA1238; the sequence has been deposited in GenBank (accession ID: PX230080).

*Male* (Figure 5c). The forewing length is 1.5 mm; the wingspan is 3.5 mm (*n* = 1). The palpi and frons are white cream. The frontal tuft is pale beige-orange. The collar and the scape are silvery white. The antenna is significantly shorter than half the length of the forewing; the flagellum is dark grey on the upper side and brownish cream on the underside, and comprises 23 segments. The thorax and the tegula are blackish grey anteriorly and silvery grey posteriorly. The forewing is silvery shiny and densely speckled with grey and dark grey scales; the forewing fascia is wide, slightly postmedian, oblique, and comprises silvery white scales. The fringe is predominantly dark grey, with a glossy white tip only at the very apex; the fringe-line is absent. The underside of the forewing is grey, without spots, androconia, or purple iridescence. The hindwing and its fringe are grey and without androconia. The legs are covered with blackish grey scales forming an interrupted marking on the upper side, and are glossy cream on the underside.

*Female*. Unknown.

*Male genitalia* (Figure 8a). The capsule is about 215 µm long and 140 µm wide. The uncus is split into two triangular lobes caudally; the gap between the lobes is narrow. The gnathos possesses two closely set, slender caudal processes arising from a slender transverse plate. The valva is 170 µm long, with two pointed apical processes; the inner lobe of the valva is slightly sinuous. The transtilla has slender and outwardly curved sublateral processes. The juxta is indistinct (membranous) or absent. The vinculum is very short but wide, truncated distally, and without lateral lobes. The phallus is 250 µm long and 60–70 µm wide basally; the vesica bears a wide band of tiny triangular and slender spine-like cornuti, the latter prevailing in the apical third; the cathrema is well developed and distinctive.

*Bionomics.* Adults fly in March; the holotype was attracted to light. Otherwise, the biology of the species is unknown.

*Distribution*. The new species is known from a single locality: Isla Zacate Grande, tropical dry forest of the Pacific coast of Honduras, at an elevation of about 10 m.

*Etymology*. The species name *pacifista* is derived from the Latinized root *paci*- (from pacificus, meaning “of or pertaining to the Pacific”) and the suffix -*ista*, commonly used to form Latin-like arbitrary names. This name acknowledges the species’ occurrence in the tropical dry forests along the Pacific coast, while also evoking associations with peace (*pax* in Latin). The name is intended to highlight both the unique ecological region where the species was discovered and the serene beauty of its natural habitat. The species epithet is treated as a feminine noun in apposition.

#### 3.1.3. Description of *Stigmella atlantidica* Stonis & Diškus, sp. nov.

*ZooBank Registration*. https://zoobank.org/NomenclaturalActs/6f96df4a-2757-43a9-bd9c-9cf326974053 (accessed on 27 August 2025).

*Material examined*. Holotype: ♂, HONDURAS, Atlantída Department, 7.5 km SE of La Ceiba, right bank of Rio Cangrejal, Hotel Rio, 100 m, 15°43ʹ37″ N, 86°44ʹ25″ W, 13 April 2023, leg. J.R. Stonis, genitalia slide no. AD1152 (MfN). Paratype: 1 ♂, the same geographical locality as holotype, 15°43ʹ34″ N, 86°44ʹ26″ W, 14 April 2023, leg. J.R. Stonis, genitalia slide no. AD1216♂ (MfN). Excluded from the type series: 1 ♀, the same locality as the male holotype, 15 April 2023, genitalia slide no. AD1217 (BRG); 1 ♀, the same label data as the male paratype (BRG).

*Diagnosis.* Externally and in the male genitalia, the new species is most similar to *S. ruralis* sp. nov. and *S. pacifista* sp. nov. (both described above). However, *S. atlantidica* sp. nov. is easily distinguished from the latter and other congeneric species by the unique set of large horn-like cornuti in the phallus, as well as by the combination of a large vinculum, bilobed uncus, and two large, pointed apical processes of the valva.

*Barcode.* Currently, no barcodes are available; our attempt to extract DNA from the male holotype and male paratype failed due to unknown causes (see Remarks).

*Male* (Figure 5d,e). The forewing length is 1.6 mm; the wingspan is 3.6 mm (*n* = 2). The palpi and the frons are cream. The frontal tuft ranges from pale orange to dark orange. The collar and the scape are glossy yellowish cream. The antenna is significantly shorter than half the length of the forewing; the flagellum is glossy brown to black on both the upper and underside, and comprises approximately 24–25 segments. The thorax, the tegula, and the forewing are densely speckled with grey-black or grey-brown scales exhibiting purple iridescence. The forewing fascia is postmedian, only slightly oblique, and comprises milky white scales. The fringe is white apically and dark brown at the tornus; the fringe-line is formed of brown-black scales. The underside of the forewing is dark greyish brown. The hindwing is densely speckled with elongated dark brown scales (holotype) or black scales with purple iridescence (paratype); some elongated dark scales extend into the fringe. It is possible that the elongated dark scales of the hindwing are androconial. The legs are black-grey on the upper side, pale grey to cream distally and on the underside. The abdomen is brown-black dorsally and cream ventrally.

*Female*. The forewing length ranges from 1.5 to 1.7 mm; the wingspan ranges from 3.4 to 3.8 mm (*n* = 2). The hindwing is grey and lacks the dark elongated scales observed in males. Otherwise, the female is similar to the male.

**Figure 9:**
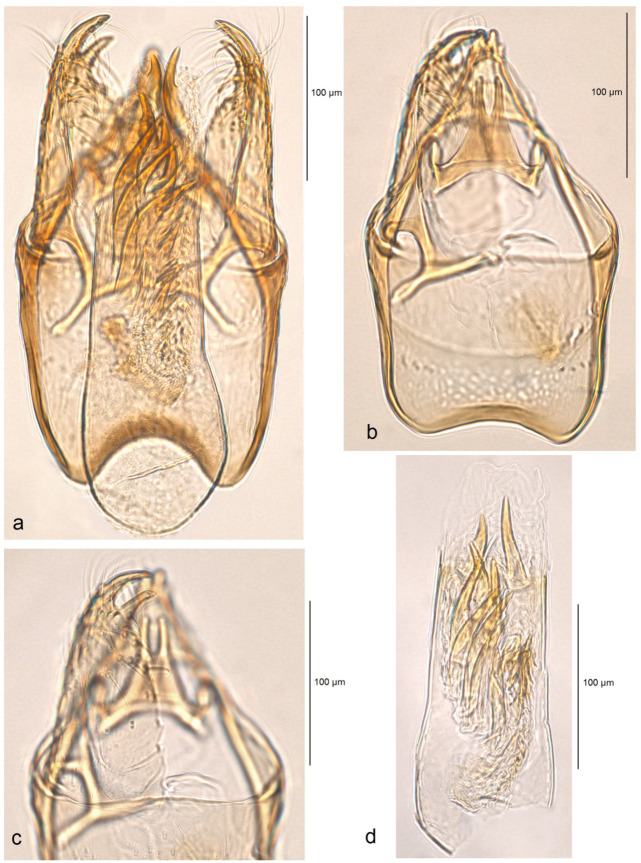
Male genitalia of *Stigmella atlantidica* Stonis & Diškus, sp. nov., from the tropical humid forests of the Atlantídica Department of Honduras: (**a**) genital capsule with phallus inside, paratype, genitalia slide no. AD1216 (MfN); (**b**,**c**) genital capsule with phallus removed, holotype, genitalia slide no. AD1052 (MfN); (**d**) phallus of the same specimen.

*Male genitalia* (Figure 9). The capsule ranges from 255 to 280 µm in length and from 155 to 160 µm in width. The uncus is large, strongly tapered distally, and divided into two lobes, each bearing two caudal papillae. The gnathos has two short caudal processes arising from a large, plate-like base. The valva is 135–150 µm long and possesses two large pointed apical processes; the inner lobe of the valva is slightly bulged. The transtilla bears stout and straight sublateral processes. The juxta is indistinctive (membranous) or absent. The vinculum is long and wide, with weakly developed anterior lobes. The phallus ranges from 250 to 270 µm in length, with the width ranging from 80 to 90 µm basally and approximately 60 µm apically. The vesica contains a wide band of spine-like and prevailing horn-like cornuti; some of the horn-like cornuti are slightly curved; the cathrema is well-developed and distinctive.

**Figure 10:**
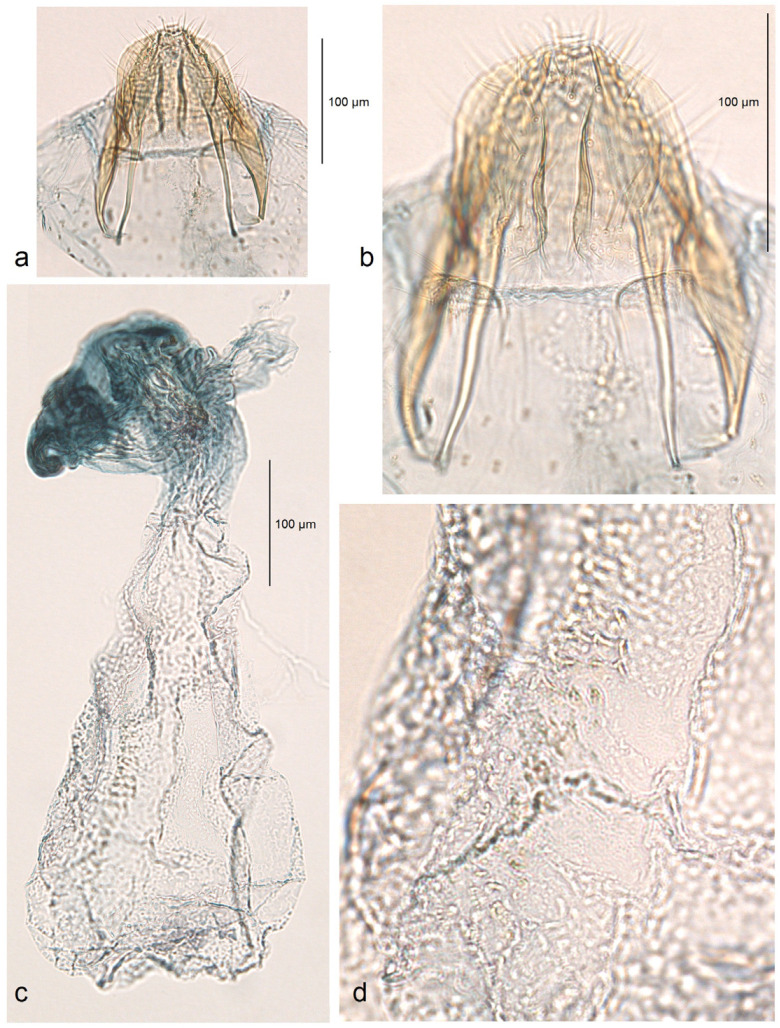
Female genitalia of *Stigmella atlantidica* Stonis & Diškus, sp. nov., from the tropical humid forests of Atlantídica Department, Honduras, genitalia slide no. AD1217 (BRG) (excluded from the type series): (**a**) ovipositor and apophyses; (**b**) the same, enlarged; (**c**) accessory sac and corpus bursae; (**d**) pectinations on the corpus bursae.

*Female genitalia* (Figure 10). The total length is approximately 730 µm. The ovipositor is short and distally rounded. The anterior and posterior apophyses are nearly equal in length; the former are wide and distally pointed, while the latter are very slender.

The accessory sac is large, irregularly rounded. The ductus spermathecae is not visible in slide no. AD1217, but is presumed to be sinuous and without distinctive coils. The corpus bursae bears comb-like and short spine-like pectinations.

*Bionomics.* Adults are active in April; the type-series males and non-type females were attracted to light. Other aspects of the species’ biology remain unknown.

*Distribution*. The species is known from a single locality on the right bank of the Río Cangrejal, approximately 7 km southeast of La Ceiba, in the Atlántida Department of Honduras, at an elevation of about 100 m.

*Etymology*. The species name *atlantidica* is derived from *Atlántida*, the name of the department in Honduras where the species was discovered, combined with the Latin suffix *-ica*, meaning “pertaining to”. This name reflects the species’ occurrence in tropical humid forests of the Atlántida Department, along the Caribbean coast of the Atlantic Ocean. The epithet highlights both the geographical and ecological context of the species’ habitat. It is treated as a feminine adjective in agreement with the genus name.

*Remarks.* Despite the lack of molecular data, *S. atlantidica* sp. nov. is a distinctive and easily diagnosed species. Two available females, collected at the same locality and time as the male holotype, resemble the males of *S. atlantidica* sp. nov. in external appearance. However, in the absence of molecular data or other conclusive evidence—and to avoid potential misidentifications—both females were excluded from the type series.

### 3.2. Case Study 2: Examination of Stigmella mutabilis sp. nov. and Its Affiliates—Species Closely Related by Morphological and Genetic Similarities and Shared Hypothetical Ancestry

During the present study, four closely related species were discovered in different, specifically selected localities in Honduras (Figure 11): *Stigmella mutabilis* Stonis & Remeikis, sp. nov. from Gracias, a mountainous valley in the Lempira Department; *S. lunacrescens* Stonis & Remeikis, sp. nov. from Tela, a tropical humid forest on the Caribbean (Atlantic) coast; *S. rostrata* Stonis & Diškus, sp. nov. from Cantarranas, in the Río Choluteca Valley of the central mountainous region; and *S. aridosa* Stonis & Remeikis, sp. nov. from tropical dry forest on the Pacific coast of Honduras. Additionally, we examined related material collected by ourselves in Colombia (*S. foreroi* Stonis & Vargas, 2019, from Departamento de Valle del Cauca) and Peru (*S. serjanica* Stonis & Diškus, sp. nov. from La Convención Province, Cerro Quintalpata). In total, the present study deals with six closely related species (Figure 12 and Figure 13). Our morphological and molecular examinations showed that all species, except *S. foreroi* Stonis & Vargas (described earlier [42]), represent new taxa. These five new species are described below.

#### 3.2.1. Description of *Stigmella mutabilis* Stonis & Remeikis, sp. nov.

*ZooBank Registration.* https://zoobank.org/NomenclaturalActs/c171e72f-1d41-4a6c-85d8-beaf939fd0aa (accessed on 27 August 2025).

*Material examined.* Holotype: ♂, HONDURAS, Lempira Department, Gracias, approx. 750 m, 14°35′35″ N, 88°34′36″ W, 6 April 2023, at light, leg. J.R. Stonis, genitalia slide no. RA1193 (MfN). *Paratypes:* 1 ♂, 5 ♀, the same label data as holotype, genitalia slide nos RA1196♀, RA1198♂, RA1200♀ (MfN); 10 ♂, 3 ♀, the same locality data as holotype, 28–31 March 2024, at light, leg. J.R. Stonis, genitalia slide nos RA1195♂, RA1199♂ (MfN); 6 ♀, the same locality data as holotype, 28–31 March 2024, mining larvae on *Serjania* sp., ex pupae 10–15 April 2024, leg. J.R. Stonis, genitalia slide no. RA1226♀ (MfN).

*Diagnosis.* Among *Stigmella* species, the deep proximal excavation of the ventral plate of the vinculum, the large, lyra-shaped thickened gnathos, the elaborated transtilla, and the large uncus with an X-shaped thickening make this species highly peculiar and distinctive. Prior to the present study, only a single resembling species—*S. foreroi* Stonis & Vargas, 2019—was known [42]. However, *S. mutabilis* sp. nov. differs from *S. foreroi* in having a basal orange spot on the forewing and in possessing cornuti in the phallus of the male genitalia (whereas *S. foreroi* lacks cornuti entirely). Molecular analysis also confirmed that the two taxa represent distinct species.

*Barcodes*. During the study, we sequenced six specimens of *S. mutabilis* sp. nov., all collected on the same day and at the same locality, and, in the case of larvae, from the same individual host-plant specimen as the paratypes of *S. mutabilis*. The sequences have been deposited in GenBank (accession IDs: PQ630806–PQ630811).

*Male* (Figure 13a,c,d). The forewing length ranges from 1.4 to 1.6 mm; the wingspan ranges from 3.2 to 3.5 mm (*n* = 12). The palpi and the frons are glossy beige cream. The frontal tuft is orange. The collar and the scape are concolorous, glossy yellowish cream. The antenna is significantly shorter than half the length of the forewing; the flagellum is brown-grey on the upper side and brownish cream on the underside, comprising approximately 24 segments. The thorax, the tegula, and the forewing are clothed with brown-black scales with weak purple iridescence. The forewing bears a transverse fascia and a large basal spot composed of beige cream or orange scales. When the wings are closed in resting position, the beige cream to yellow-orange spot on the forewing forms a large dorsal marking that resembles a full moon—a feature characteristic of most males and especially prominent in females. The fascia is slightly postmedian and consists of silvery shiny scales, though it may appear golden glossy under certain angles or lighting. The fringe is white apically and grey on the tornus. The underside of the forewing is densely irrorated with black scales, some with purple iridescence; androconia are absent. The hindwing and its fringe are grey and without spots or androconia. The legs are glossy greyish cream on the underside, but densely covered with brown-black to black scales on the upper side. The abdomen is brown-black dorsally and shiny silvery ventrally; the anal tufts are indistinct or absent; the genital plates are clothed with beige cream scales.

*Female* (Figure 13b). The forewing length ranges from 1.3 to 1.6 mm; the wingspan ranges from 3.0 to 3.4 mm (*n* = 8). The beige cream or orange spot on the forewing—visible as a dorsal spot when the wings are folded in resting position—tends to be larger and more vivid than in males. Otherwise, females are similar to males.

**Figure 14:**
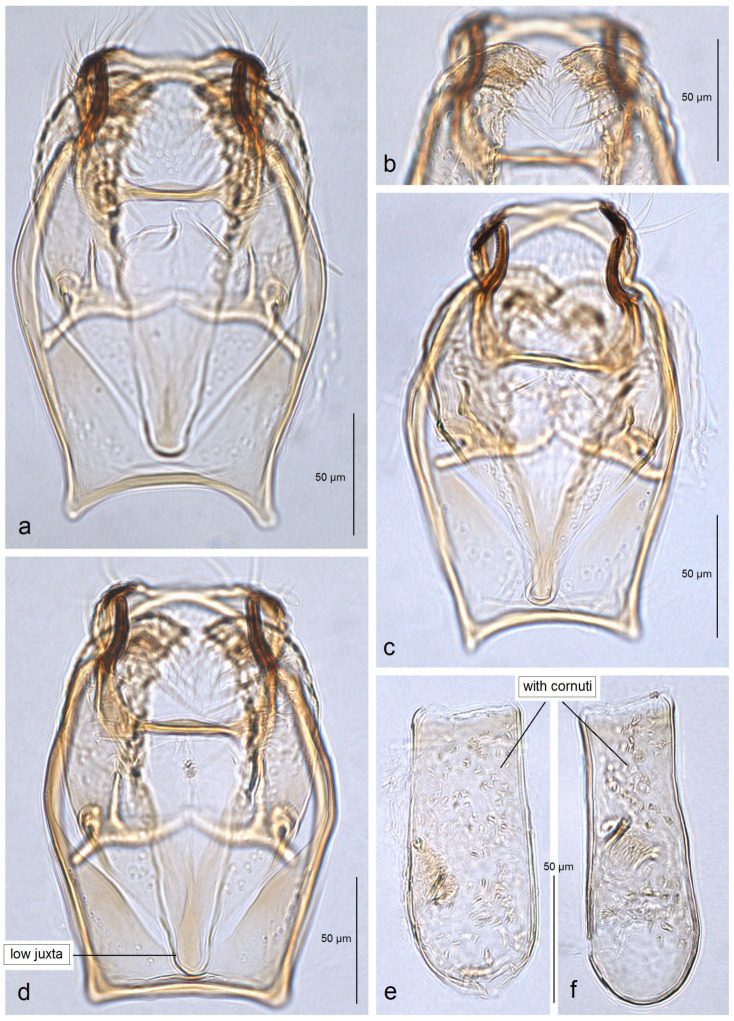
Male genitalia of *Stigmella mutabilis* Stonis & Remeikis, sp. nov., from Gracias, a mountainous valley in Lempira Department, Honduras: (**a**,**b**) genital capsule with phallus removed, paratype, genitalia slide no. RA1195 (MfN); (**c**) the same, holotype, slide no. RA1193 (MfN); (**d**) the same, paratype, slide no. RA1198 (MfN); (**e**) phallus, holotype, slide no. RA1193 (MfN); (**f**) the same, paratype, slide no. RA1198 (MfN).

*Male genitalia* (Figure 14). The genital capsule ranges from 170 to 190 µm in length and 120 to 140 µm in width. The uncus is very large, bearing an X-shaped thickening with weakly defined lateral lobes that appear darkened sublaterally. The gnathos is large and lyra-shaped, with darkened caudal processes. The valva is inwardly curved and pointed apically. The transtilla is elaborated, with a sinuous anterior margin. The juxta is weakly thickened, two-folded, and positioned very low—it reaches the anterior margin of the vinculum. The vinculum exhibits an extremely deep posterior excavation, which accommodates the deeply set juxta. The phallus ranges from 120 to 140 µm in length and 40 to 50 µm in width; it lacks apical lateral thickenings. The vesica contains numerous small, triangular, weakly chitinized cornuti that are scattered rather than collected into a band. The cathrema is weakly developed and may be indistinct.

**Figure 15:**
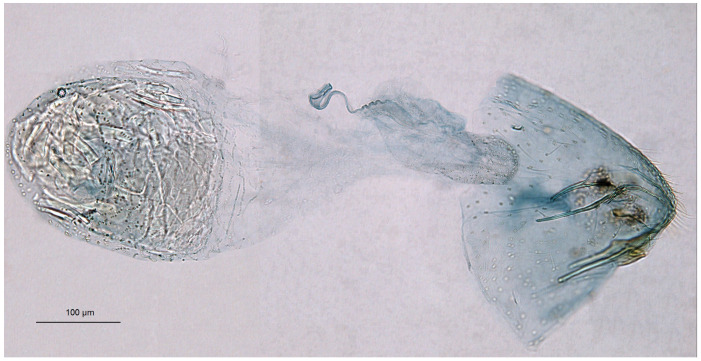
Female genitalia of *Stigmella mutabilis* Stonis & Remeikis, sp. nov., from Gracias, a mountainous valley in Lempira Department, Honduras, genitalia slide no. RA1195, paratype (MfN).

*Female genitalia* (Figure 15). The total length of the genitalia ranges from 610 to 760 µm. The abdominal apex is triangular in shape, and the ovipositor is only weakly developed. The anterior apophyses are slightly to significantly shorter than the posterior apophyses; the posterior apophyses are very slender, while the anterior ones are wider basally. The accessory sac is large, elongated, and weakly folded. The ductus spermathecae is relatively short and sinuous, with approximately four minuscule coils and one or two partially developed larger coils. The corpus bursae is large and bulbous, with relatively long, comb-like pectinations.

**Figure 16:**
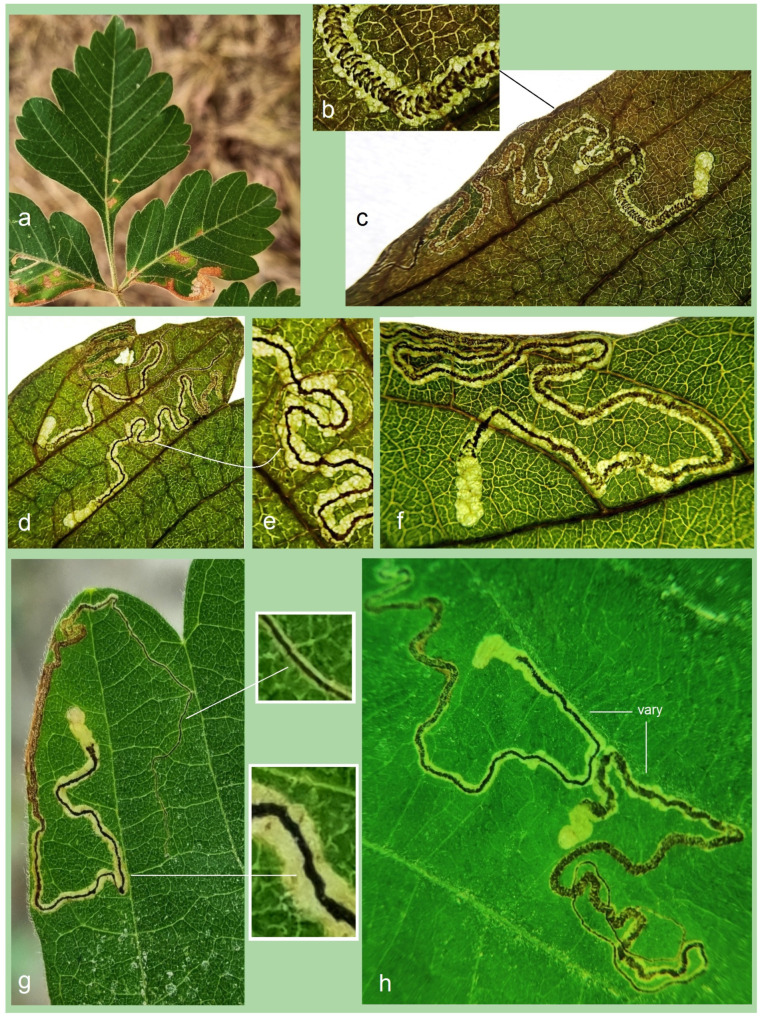
Bionomics of *Stigmella mutabilis* Stonis & Remeikis, sp. nov.: (**a**) *Serjania* sp. (Sapindaceae), the host plant; (**b**–**h**) variable leaf mines, from which *S. mutabilis* sp. nov. was reared, collected in Gracias, a mountainous valley in Lempira Department, Honduras (see *Remarks*).

**Figure 17:**
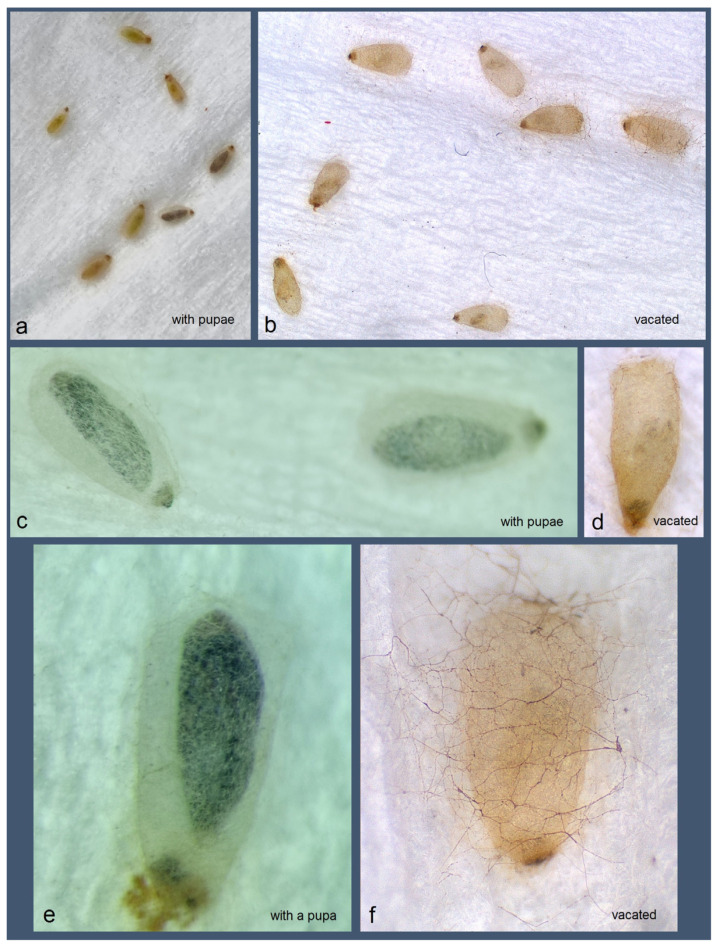
Transparent (atypical) cocoons of *Stigmella mutabilis* Stonis & Remeikis, sp. nov., from Gracias, a mountainous valley in Lempira Department of Honduras: (**a**,**c**,**e**) with pupae inside; (**b**,**d**,**f**) vacated cocoons, without pupae.

*Bionomics.* The host plant is *Serjania* sp. (Sapindaceae) (Figure 16a). The larvae mine the leaves. Mining larvae were found in high numbers during late March and early April. The leaf mines are very long and slender, sinuous, with variably deposited frass (Figure 16b–h) (see Remarks). Before pupation, larvae spin unusual, almost transparent, silky cocoons (Figure 17). In the field, adults were observed flying from March to early April; in the laboratory, adults hatched in mid-April. The majority of type-series specimens were attracted to light traps. Otherwise, the biology of the species remains unknown.

*Distribution.* Known only from the single locality: Gracias, Lempira Department, at an elevation of about 750 m.

*Etymology.* The species name *mutabilis* is derived from the Latin adjective meaning “changeable” or “variable”, referring to the variability of the leaf mines and the highly variable external appearance of the adults. The forewings of *S. mutabilis* sp. nov. exhibit a range of patterns, from being nearly entirely dark with only a slender pale transverse fascia, to a combination of this fascia with a large orange-yellow spot on the dorsum. The Latin adjective *mutabilis* is of common gender and, in this case, is treated as feminine.

*Remarks.* Theoretically, the presence of highly variable leaf mines on the same host plant at the same time could suggest larvae of two species mining simultaneously. However, in practice, all reared material, specimens collected by light trap near the same host-plant specimen, plant bearing different leaf mines, and randomly selected larvae for DNA sequencing have consistently yielded a single species, *S. mutabilis* sp. nov.

#### 3.2.2. Description of *Stigmella lunacrescens* Stonis & Remeikis, sp. nov.

*ZooBank Registration.* https://zoobank.org/NomenclaturalActs/e9415cee-85d1-4e89-907e-080e48fe0e03 (accessed on 27 August 2025).

*Material examined.* Holotype: ♂, HONDURAS, Atlantída Department, Tela (3 de Mayo), 10–30 m, 15°45′56”–15°46′09″ N, 87°27′06”–87°23′15″ W, 2–19 April 2024, at light, leg. J.R. Stonis, genitalia slide no. RA1202 (MfN). Paratypes: 1 ♂, the same label data as holotype, at light, leg. J.R. Stonis (MfN); 2 ♂, Fransisco Morazán Department, Cantarranas (=San Juan de Flores), 660–690 m, 14°15′50”–14°16′18″ N, 87°01′31″ W, 10–15 February 2024, at light, leg. J.R. Stonis, genitalia slide no. RA1213 (MfN). Excluded from the type series: 1 ♀, Fransisco Morazán Department, Cantarranas, 660–690 m, 14°15′50”–14°16′17″ N, 87°01′35”–87°01′07″ W, 10–15 February 2024, at light, leg. J.R. Stonis (BRG); 1 ♀ Atlantída Department, Tela (3 de Mayo), 10–30 m, 15°45′56”–15°46′09″ N, 87°27′06”–87°23′15″ W, 2–19 April 2024, at light, leg. J.R. Stonis, genitalia slide no. RA1225♀ (BRG). Also studied: vacated leaf mines collected on *Serjania* sp., Atlantída Department, Tela (3 de Mayo), 10–30 m, 15°45′56”–15°46′09″ N, 87°27′06”–87°23′15″ W, 25 April 2025 (BRG).

*Diagnosis. Stigmella lunacrescens* sp. nov. is similar to *S. mutabilis* sp. nov. (described above); however, *S. lunacrescens* differs from the latter by its dark forewing lacking a basal orange spot and by the absence of cornuti in the phallus (whereas *S. mutabilis* has numerous tiny triangular cornuti scattered on the vesica of the phallus). DNA sequence data also clearly indicate that *S. lunacrescens* and *S. mutabilis* are two distinct species.

*Barcodes.* We barcoded three specimens: one specimen from Cantarranas, Morazán Department, collected on 11 February 2024, and two specimens from Tela (3 de Mayo), Atlantída Department, collected on 19 April 2024; their sequences have been deposited in GenBank (accession IDs: PQ630812–PQ630814).

*Male* (Figure 12a). The forewing length ranges from 1.4 to 1.5 mm; the wingspan ranges from 3.2 to 3.4 mm (*n* = 3). The palpi and the frons are golden cream. The frontal tuft is orange. The collar and the scape are glossy yellowish cream. The antenna is shorter than half the length of the forewing; the flagellum is glossy black-grey with purple iridescence on the upper side, dark grey on the underside, comprising about 19 segments. The thorax and the tegula are dark, covered with black-brown scales. The forewing is covered with dark black-brown scales showing purple iridescence; at certain angles, the forewing may appear bronzy-brown or golden glossy dark brown. The forewing fascia is nearly median and straight transverse (almost no obliqueness), composed of silvery shiny or, at certain angles, golden shiny scales. The fringe is white apically, dark grey to black-grey on the tornus. The forewing underside is black-grey with weak purple iridescence, without androconia. The hindwing and its fringe are dark grey, without spots or androconia. The legs are glossy cream to grey; the hindlegs are covered with brown-black scales showing purple iridescence dorsally and ventrally, except for the pale tarsi. The abdomen is brown-black dorsally, silvery shiny ventrally; the anal tufts are indistinct or absent; the genital plates (segments) contrast with the abdomen color, being covered with beige-cream to yellow-beige scales.

*Female*. The forewing length ranges from 1.5 to 1.6 mm; the wingspan ranges from 3.3 to 3.5 mm (*n* = 2). Otherwise, females are similar to males.

**Figure 18:**
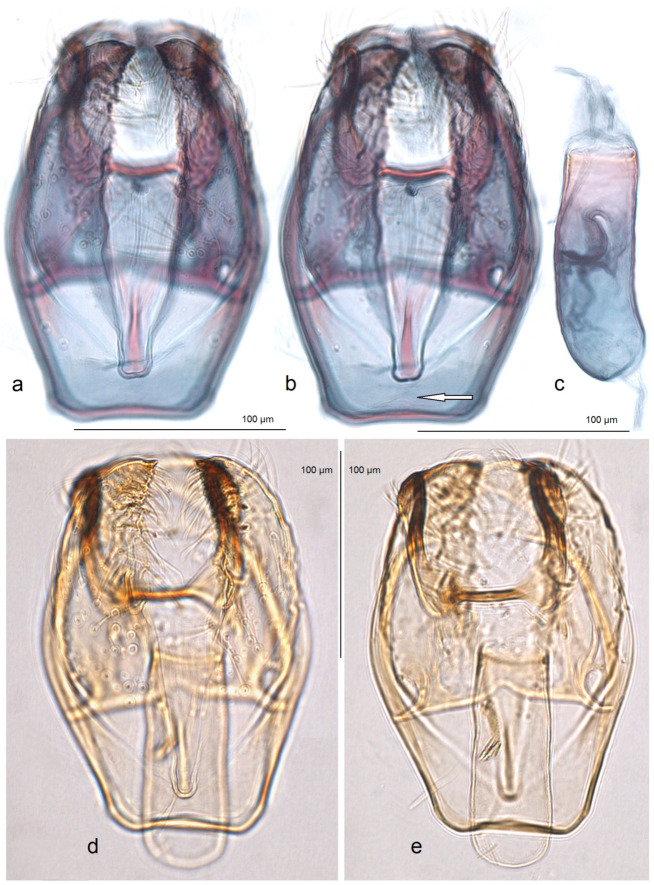
Male genitalia of *Stigmella lunacrescens* Stonis & Remeikis, sp. nov: (**a**–**c**) paratype, from Cantarranas, Francisco Morazán Department, genital capsule with phallus removed, genitalia slide no. RA1213 (MfN); (**d**,**e**) holotype, from Tela, Atlantída Department, genital capsule with phallus inside, genitalia slide no. RA1202 (MfN).

*Male genitalia* (Figure 18). The genital capsule ranges from 150 to 185 µm in length and from 120 to 130 µm in width. The uncus is very large, with an X-shaped thickening and weakly demarcated lobes; the sublateral corners of the uncus lobes are darkened. The gnathos is large and lyra-shaped, with darkened caudal processes and stout lateral corners. The valva is slightly inwardly curved and pointed apically. The transtilla is elaborated, with a sinuous anterior margin. The juxta is weakly thickened, folded, and bears a thickened, 40 µm long median element. Its position is not as maximally low as in *S. mutabilis* sp. nov.—the juxta does not reach the anterior margin of the vinculum. The vinculum has a deep posterior excavation, but it does not reach the anterior rim. The phallus ranges from 115 to 120 µm in length and is about 40 µm wide, lacking apical lateral thickenings. The vesica bears no cornuti (which are characteristic of *S. mutabilis* sp. nov.). The cathrema is variably developed and sometimes rather indistinct.

**Figure 19:**
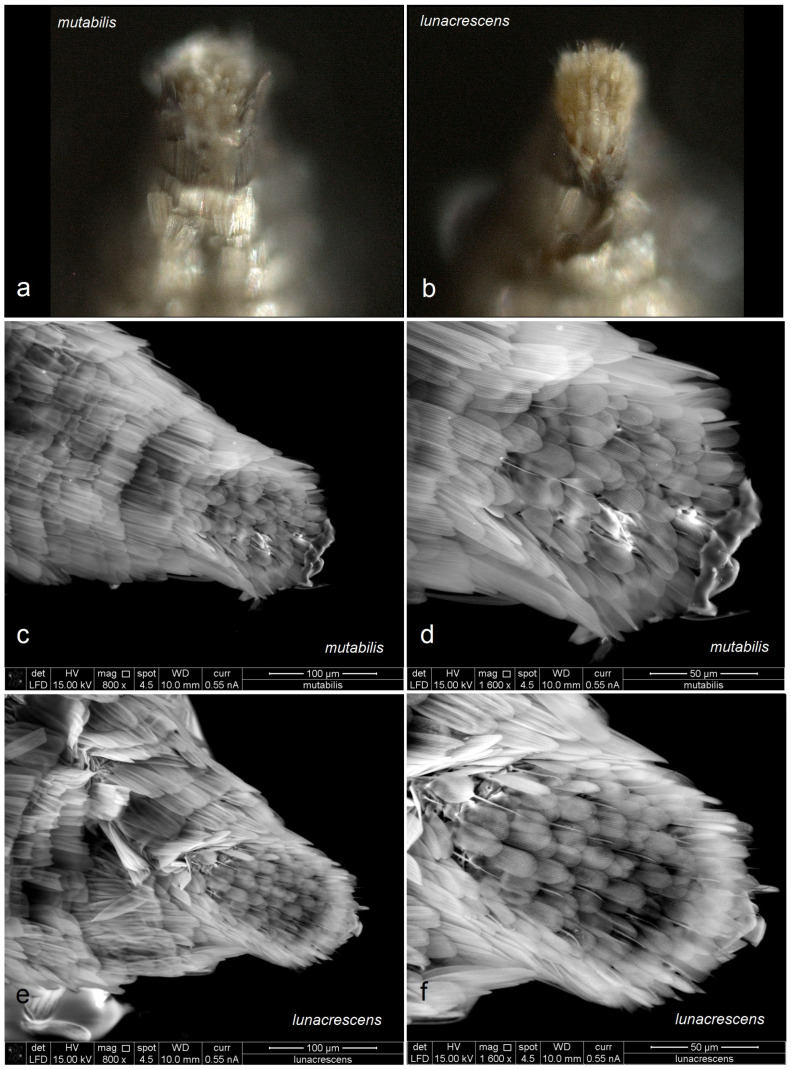
Comparison of the female ovipositor of two related *Stigmella* Schrank species: (**a**,**c**,**d**) S. *mutabilis* Stonis & Remeikis, sp. nov.; (**b**,**e**,**f**) *S. lunacrescens* Stonis & Remeikis, sp. nov. *Note*: The images (**a**,**b**) are Leica microscope photographs; the images (**c**–**f**) are SEM micrographs.

*Female genitalia* (see Remarks). The total length is approximately 640 µm. The abdominal apex is angular but rounded caudally, and the ovipositor is weakly developed. For comparison of the ovipositor of *S. lunacrescens* with that of the related *S. mutabilis*, see Figure 19. The anterior apophyses are slightly or significantly shorter than the posterior apophyses; the latter are very slender, while the anterior ones are wide in the proximal half. The accessory sac is large, elongated, and slightly folded. The ductus spermathecae is relatively short and sinuous, with a couple of coils and a vesicle (but poorly visible in genitalia slide no. RA1225, MfN). The corpus bursae is bulbous; the pectinations are either indistinct or absent (also poorly visible in genitalia slide no. RA1225, MfN).

**Figure 20:**
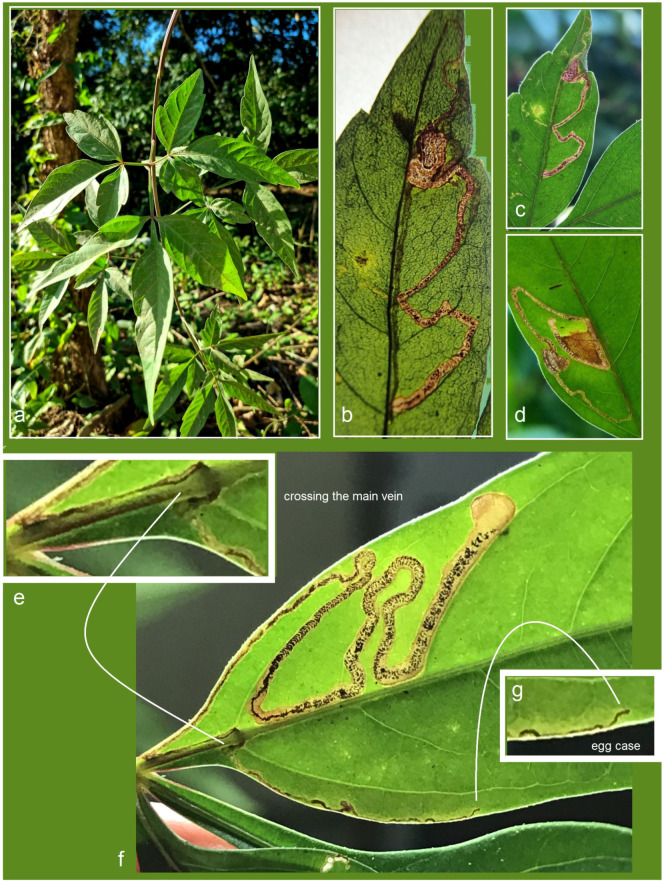
Bionomics of *Stigmella lunacrescens* Stonis & Remeikis, sp. nov.: (**a**) *Serjania* sp. (Sapindaceae), a host plant; (**b**–**g**) leaf mines, sampled in Tela, Atlantída Department, Honduras.

*Bionomics* (Figure 20). The host plant is *Serjania* sp. (Sapindaceae) (Figure 20a), though this association has not yet been confirmed through larval rearing (see Remarks). Vacated leaf mines were found in high numbers in April. The mines are very long, slender, and sinuous, with variably deposited frass arranged in close-set arcs or loosely scattered (Figure 20f). Based on the examination of vacated mines, it was observed that young feeding larvae can cross the main vein of the leaf (Figure 20e). Adults were observed flying in April and were attracted to light. Other aspects of the species’ biology remain unknown.

*Distribution.* Known from two localities in northern coastal and central mountainous Honduras: Tela (3 de Mayo), Atlantída Department, at an elevation of about 10–30 m, and Cantarranas, Francisco Morazán Department, at an elevation of about 650–690 m.

*Etymology.* The name of the new species is derived from the Latin *luna crescentis*, meaning “crescent moon”, referring to the moths collected at night that possess dark forewings with a slender but shiny fascia. Unlike the closely related *S. mutabilis* sp. nov., this species lacks the orange spot reminiscent of a full moon. The name *lunacrescens* is an adjective and is treated here as feminine.

*Remarks.* All available females were excluded from the type series due to uncertainty and lack of molecular or other confirmatory evidence. Although all material was collected at light (including the type series specimens), *Serjania* sp. is assumed to be the host plant because the light trap was set near numerous vacated leaf mines on *Serjania*. These long, sinuous leaf mines resemble those of the related *S. mutabilis* sp. nov., which has been confirmed as a *Serjania* leaf miner.

#### 3.2.3. Description of *Stigmella aridosa* Stonis & Remeikis, sp. nov.

*ZooBank Registration*. https://zoobank.org/NomenclaturalActs/ca07ea39-8fbe-4f2a-a60d-0cd04502ff82 (accessed on 27 August 2025).

*Material examined.* Holotype: ♂, HONDURAS, Pacific coast, Isla del Tigre, Amapala, approx. 10 m, 13°17′41″ N, 87°38′41″ W, 17 March 2023, at light, leg. J.R. Stonis, genitalia slide no. RA1231 (MfN). Excluded from the type series: 1 ♀, San Lorenzo, 1.5 km eastwards along the Pan American Hwy (left side), approx. 40 m, 13°25′59″ N, 87°25′24″ W, 6–7 February 2023, at light, leg. J.R. Stonis, genitalia slide no. RA1205 (BRG); 1 ♀, Pacific coast, Isla Zacate Grande, El Moray (Restaurante Terra Mar), 20 m, 13°21′28″ N, 87°36′06″ W, 15–16 February 2023, at light, leg. J.R. Stonis, genitalia slide RA1206 (BRG).

*Diagnosis.* By having pale scales on the basal third of the forewing (i.e., a dorsal spot visible when the wings are closed in the resting position), *Stigmella aridosa* sp. nov. is most similar to *S. mutabilis* sp. nov. (described above). However, *S. aridosa* sp. nov. differs from the latter by the uniquely rounded shape of the male genitalia capsule and by the absence of cornuti in the phallus (*S. mutabilis* sp. nov. has numerous tiny triangular cornuti on the vesica).

*Barcodes.* Molecular data are currently unavailable.

*Male*. The forewing length is about 1.3 mm; the wingspan is 2.9 mm (*n* = 1). The palpi and the frons are silvery shiny. The frontal tuft is pale orange. The collar and the scape are silvery shiny. The antenna is significantly shorter than half the length of the forewing; the flagellum is pale yellowish beige with some brown-black scales on the upper side, comprising about 25 segments. The thorax and the tegula are covered with pale brown, silvery glossy scales, with an admixture of some dark scales. The basal third of the forewing is pale brown, silvery glossy (note that females have yellow cream scales), and, as in *S. mutabilis* sp. nov., forms a large dorsal spot when both wings are closed in the resting position. The transverse fascia of the forewing is wide and straight, comprising white, silvery shiny scales; the areas before the fascia and on the forewing apex are densely irrorated with blackish brown scales with golden gloss. The fringe is glossy brownish grey; the fringe line is absent. The forewing underside is glossy grey with some purple iridescence, without androconia. The hindwing and its fringe are pale brown-grey, without spots or androconia. The legs are glossy beige cream on the underside but covered with dark grey scales on the upper side.

*Female* (Figure 12c). The forewing length ranges from 1.3 to 1.6 mm; the wingspan ranges from 3.0 to 3.6 mm (*n* = 2). Females are much brighter than males, with a distinct yellow cream spot on the basal third of the forewing (or a dorsal spot when wings are closed in resting position); such a female (not a male) is illustrated in Figure 12c. Otherwise, females are similar to males.

*Male genitalia* (Figure 21). The genital capsule is distinctly oval-shaped (rounded), about 205 µm long and 140 µm wide. The uncus is very large, with an X-shaped thickening and weakly separated lateral lobes; the lobes are thickened and darkened laterally.

The gnathos is large, lyra-shaped, with darkened caudal processes. The valva is slightly inwardly curved and almost pointed at the apex. The transtilla is elaborated, with a sinuous anterior margin but very short sublateral processes. The juxta is weakly thickened, folded, with a spine-like median element; its position is very low, reaching the anterior rim of the vinculum. The vinculum is very small, with an extremely deep posterior excavation engaged by the valvae and the deeply positioned juxta. The phallus is about 130 µm long, without lateral thickenings apically; the vesica is without cornuti; the cathrema is well chitinized and distinctive.

*Female genitalia.* The total length is unknown, as some parts are probably missing (torn in the available genitalia slides). The abdominal apex is triangularly shaped; the ovipositor is weakly pronounced. The anterior apophyses are slightly shorter than the posterior apophyses; the latter are slender, while the anterior ones are wide in the proximal half. The accessory sac is large, rounded, and weakly folded. The ductus spermathecae is sinuous, without pronounced coils. The corpus bursae is probably missing in slide no. RA1206 (BRG); therefore, the female genitalia are not illustrated here.

*Bionomics.* Based on the observation of vacated leaf mines resembling those of *S. mutabilis* sp. nov., which are abundant at the light-trap collecting site of *S. aridosa* sp. nov., the host plant is presumed to be a *Serjania* species (Sapindaceae). However, no rearing records are yet available. Adults fly in February and can be attracted to light. Otherwise, the biology of the species remains unknown.

*Distribution.* The new species is currently known from a few localities within tropical dry forests along the Pacific coast of Honduras (Isla del Tigre, San Lorenzo, and Isla Zacate Grande), at elevations ranging from 10 to 40 m.

*Etymology.* The specific epithet *aridosa* is derived from the Latin adjective *aridus* (dry), with the suffix *-osa* added to convey an association with dryness. The name was chosen to reflect the species’ ecological association, as it was discovered in tropical dry forest habitats and appears to be active during the dry season. This name highlights the species’ adaptation to arid environments and its seasonal behavior in response to dry conditions.

#### 3.2.4. Description of *Stigmella rostrata* Stonis & Diškus, sp. nov.

*ZooBank Registration.*https://zoobank.org/NomenclaturalActs/fdc7aa99-e2ae-45bf-a0ca-461ec6377aaa (accessed on 27 August 2025).

*Material examined.* Holotype: ♂, HONDURAS, Francisco Morazán Department, Cantarranas (=San Juan de Flores), ca. 660 m, 14°15′57″ N, 87°01′31″ W, 18–23 April 2023, at light, leg. J.R. Stonis, genitalia slide no. AD1203 (MfN).

*Diagnosis.* The new species most closely resembles the Honduran species *Stigmella mutabilis* sp. nov., *S. lunacrescens* sp. nov., and *S. aridosa* sp. nov. (described above), as well as the Colombian *S. foreroi* Stonis & Vargas, 2019 [42]. However, *S. rostrata* sp. nov. differs from all these species by the presence of a spine-like apical process on the valva, a wide juxta, and the distinctly rectangular shape of the male genital capsule.

*Barcode*. One specimen collected in Cantarranas, Morazán Department, Honduras, on 11 February 2024 (not part of the type series) was barcoded, and its sequence has been deposited in GenBank (accession ID: PQ630815). However, the genitalia of this specimen were not dissected or examined prior to preservation in alcohol for DNA extraction. As a result, there is some uncertainty regarding the identity of the sequenced specimen and the reliability of the barcode.

*Male* (Figure 12b). The forewing length is 1.3 mm; the wingspan is approximately 3.0 mm (*n* = 1). The palpi and the frons are greyish cream. The frontal tuft is pale orange. The collar and scape are silvery shiny. The antenna is significantly shorter than half the length of the forewing; the flagellum is glossy pale brownish grey, comprising approximately 19 segments. The thorax, the tegula, and the basal half of the forewing are covered with pale brownish grey, silvery glossy scales. The forewing bears a slender, silvery white transverse fascia; the apex is densely irrorated with black scales. The fringe is whitish apically and dark grey to blackish grey on the tornus. The underside of the forewing is dark grey with some purple iridescence, without androconia. The hindwing and its fringe are grey, without spots or androconia. The legs are glossy pale grey on the underside, but with dark grey scales and some purple iridescence on the upper side.

*Female.* Unknown (see Remarks).

*Male genitalia* (Figure 22). The genital capsule is 160 µm long and 60 µm wide. The uncus is very large, with an X-shaped thickening and weakly separated lateral lobes; the distal corners of the lobes are darkened. The gnathos is large and lyra-shaped, with darkened caudal processes.

The valva is slightly bulged in the apical third and bears an inwardly bent beak-shaped thickening (an apical process). The transtilla is elaborated, with a slightly sinuous anterior margin. The juxta is weakly thickened and wide, with two very short but wide caudal lobes. The vinculum has two triangular lateral lobes and an extremely deep posterior excavation, reaching up to the anterior rim. The phallus is about 110 µm long, lacking lateral thickenings apically; the vesica bears no cornuti; the cathrema is well-developed and distinctive.

*Bionomics.* The host plant is unknown. Adults have been observed flying in April. Otherwise, the biology of the species remains unknown.

*Distribution.* Known only from the single locality of Cantarranas, Morazán Department, Honduras, at an elevation of approximately 660 m.

*Etymology.* The species name *rostrata* is derived from the Latin adjective *rostratus*, meaning “beaked” or “having a beak”, which in turn comes from *rostrum*, meaning “beak” or “snout.” This name refers to the distinctive apical process of the male valva, which is shaped like an inwardly bent beak or hook. The characteristic beak-shaped valval thickening is a key diagnostic feature distinguishing this species from its congeners.

*Remarks.* During our study, a few females resembling *S. rostrata* sp. nov. were collected at the same locality as the holotype. However, subsequent dissections revealed that their genitalia did not fully correspond to those of species in the *S. mutabilis* group. Due to uncertainties regarding the taxonomic position of these females, they were excluded from further analysis.

#### 3.2.5. The Colombian *Stigmella foreroi* Stonis & Vargas, 2019

*Material examined*. Holotype: ♂, COLOMBIA, Departamento de Valle del Cauca, Municipio de Dagua, El Naranjo, 550 m, at light, 3°46′46″ N, 76°43′36″ W, 21–23 February 2019, leg. J.R. Stonis & S.A. Vargas, genitalia slide no. RA1026 (PUJ). Paratype: 1 ♂, the same data as holotype (PUJ).

*Barcode*. We barcoded one male specimen of *Stigmella foreroi* with the same collecting data as the holotype. The sequence has been deposited in GenBank (accession ID: MN982355).

*Remarks.* The male forewing length ranges from 1.4 to 1.6 mm; the wingspan ranges from 3.2 to 3.6 mm (*n* = 2) (Figure 12d). Females are unknown. The male genital capsule measures approximately 200 µm in length and 125 µm in width; the phallus is about 125 µm long and bears apical lateral thickenings. *S. foreroi* is currently known only from the type locality: El Naranjo, Valle del Cauca, southwestern Colombia, at an elevation of about 550 m. The habitat is the tropical humid forest situated on the eastern edge of the Chocó biogeographical province. The species was originally described and illustrated in detail in 2019 [42]; moreover, genitalia illustrations can also be found in the most recent monograph on Neotropical Nepticulidae [10].

#### 3.2.6. Description of *Stigmella serjanica* Stonis & Diškus, sp. nov., a *Serjania*-Feeding Species from Peru

*ZooBank Registration*. https://zoobank.org/NomenclaturalActs/f71a3df2-7656-4b49-9c25-0d1eb1082890 (accessed on 27 August 2025).

*Material examined.* Holotype: ♂, PERÚ, Cusco Region, La Convención Province, Maranura, 1220 m, 12°57′56” S, 72°39′18″ W, 21 June 2018, feeding larvae on *Serjania* sp. (Sapindaceae), ex pupa 3 July 2018, field card no. 5277, leg. J.R. Stonis, A. Diškus & S. Arotaype-Puma, genitalia slide no. AD1222 (MfN). Paratype: 1 ♂, the same label data as holotype, genitalia slide no. AD1148 (from adult in pupal skin; no pinned moth preserved) (MfN).

*Diagnosis.* Among *Stigmella* species, *S. serjanica* sp. nov. is distinguished by a unique combination of morphological features: an apically rounded, inwardly bent valva; a large, angular uncus; and a lyra-shaped, thickened gnathos. It differs from the most resembling species—Honduran *S. mutabilis* Stonis & Remeikis, sp. nov., *S. lunacrescens* Stonis & Remeikis, sp. nov., *S. aridosa* Stonis & Remeikis, sp. nov., *S. rostrata* Stonis & Diškus, sp. nov. (described above), and the Colombian *S. foreroi* Stonis & Vargas, 2019 [42]—by the presence of a distinct band of spine-like cornuti in the phallus, a posteriorly unexcavated vinculum with triangular lateral lobes anteriorly, a basally truncated valva, and the uniquely rounded apex of the valva. Molecular analysis of *S. serjanica* sp. nov. further confirms the distinctiveness of this new species.

*Barcode*. We barcoded one specimen collected in Maranura, La Convención Province, Peru, on 21 June 2018. The sequence has been deposited in GenBank (accession ID: PQ630816).

*Male* (Figure 12e). The forewing length is 1.6 mm; the wingspan is 3.5 mm (*n* = 1). The palpi and frons are cream. The frontal tuft is intense beige-orange. The collar and scape are glossy yellowish cream. The antenna is significantly shorter than half the length of the forewing; the flagellum is grey-brown with golden gloss and some purple iridescence on the upper side, pale grey on the underside (the number of segments is unknown). The thorax and the tegula are covered with blackish brown scales. The forewing is dark brown-black with purple iridescence, but may appear paler at certain angles. The fascia is slightly postmedian (or almost subapical), comprising silvery shiny scales; sometimes, at certain angles or under specific illumination, the fascia may look slightly yellowish. The fringe is glossy grey. The underside of the forewing is dark brown with some purple iridescence, without androconia. The hindwing and its fringe are brown, without spots or androconia. The legs are glossy grey; the hindlegs are covered with grey-black scales on the upper side, but the tarsi are orange cream. The abdomen is blackish grey on the upper side, glossy pale grey on the underside; the anal tufts are indistinct or absent; the genital segments are covered with grey cream scales.

*Female.* Unknown.

**Figure 23:**
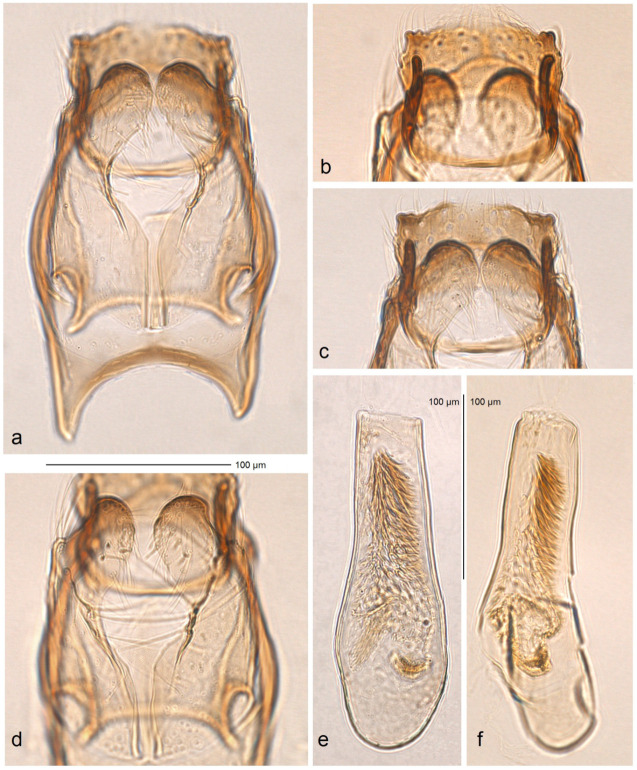
Male genitalia of *Stigmella serjanica* Stonis & Diškus, sp. nov., from Maranura, La Convención Province, Peru, 1220 m: (**a**,**c**) capsule with phallus removed, holotype, genitalia slide no AD1222 (MfN); (**b**,**d**) the same, paratype, genitalia slide no. AD1148 (MfN); (**e**) phallus, holotype AD1222 (MfN); (**f**) the same, paratype AD1148 (MfN).

*Male genitalia* (Figure 23). The genital capsule ranges from 220 to 230 µm in length and from 125 to 130 µm in width. The uncus is very large and angular; the X-shaped thickening is absent; the uncus lobes are thickened but not darkened laterally. The gnathos is large, lyra-shaped, with slightly darkened caudal processes. The valva is inwardly bent, distinctly rounded apically, and truncated basally. The transtilla consists of a straight transverse bar (not sinuous); sublateral processes of the transtilla are short. The juxta is membranous (invisible) or absent. The vinculum lacks posterior excavation and has triangular lateral lobes anteriorly. The phallus ranges from 180 to 190 µm in length and 30 to 60 µm in width, without lateral thickenings apically; the vesica possesses a band of spine-like cornuti; the cathrema is well thickened and distinctive.

**Figure 24:**
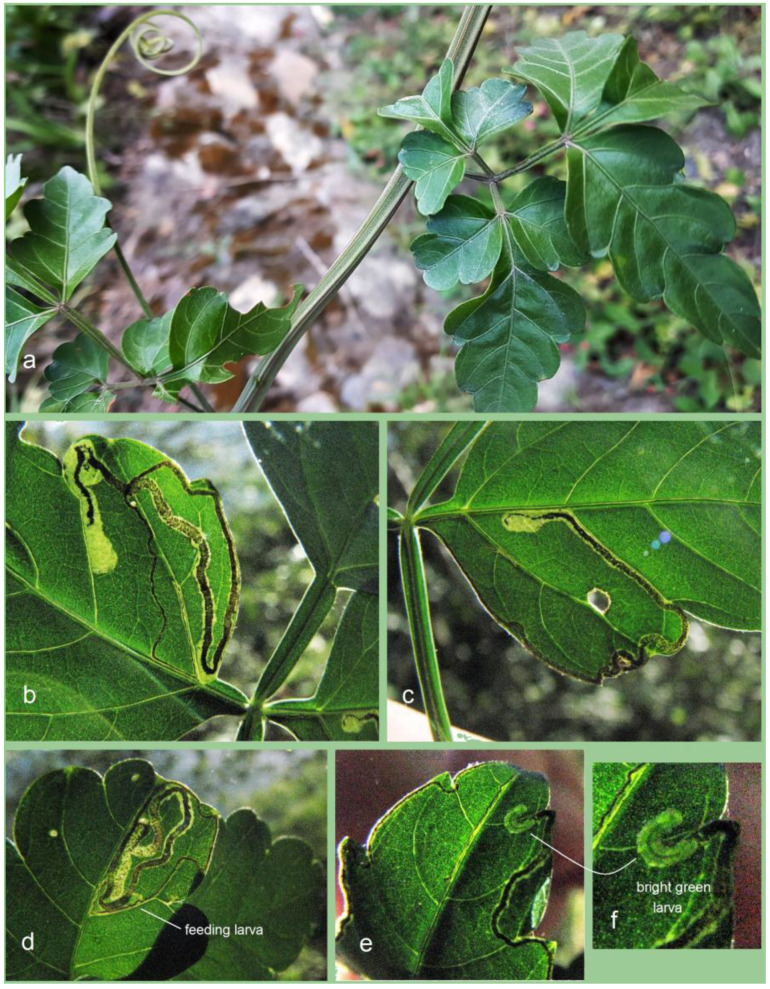
Bionomics of *Stigmella serjanica* Stonis & Diškus, sp. nov., La Convención Province, Peru, 1220 m: (**a**) host plant, *Serjania* sp. (Sapindaceae); (**b**–**f**) leaf mines.

*Bionomics* (Figure 24). The host plant is *Serjania* sp. (Sapindaceae) (Figure 24a). The larvae mine the leaves. Mining larvae were detected in late June. The leaf mines are long, slender, and sinuous, with a relatively wide line of frass mostly deposited in very close-set (compact) arcs; clear margins of the mine track are narrow (Figure 24b–f). The larvae are bright green with a dark green intestine. The adults were reared under laboratory conditions in early July. The mining was observed in a shaded area near plantations and orchards, on host plant branches hanging 1.5–1.8 m above the ground. Otherwise, the biology of the species is unknown.

*Distribution.* Known only from a single locality: Maranura, La Convención Province, Perú, at an elevation of about 1220 m.

*Etymology.* The new species name derives from the host plant genus name *Serjania* Mill.

#### 3.2.7. Molecular Considerations of the *Stigmella mutabilis* sp. nov. and Related Species

**Figure 25:**
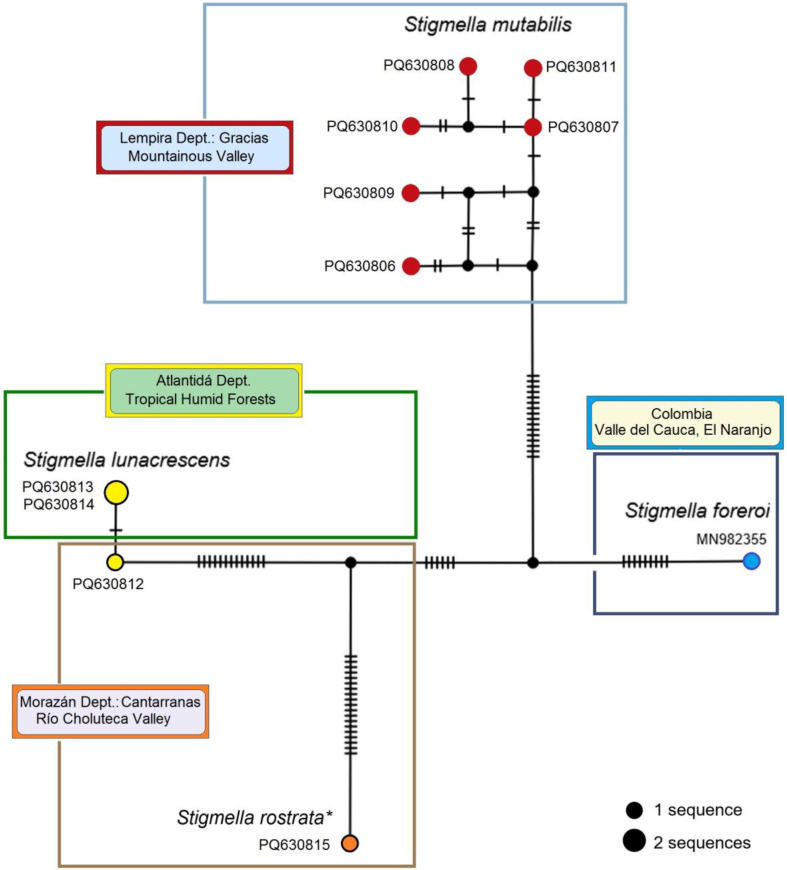
Mitotype network of 657 bp-long mtDNA CO1-5′ sequences of *Stigmella mutabilis* sp. nov. and related species, constructed using the TCS Network algorithm. Circle size is proportional to the frequency of each mitotype. Smaller, unnamed circles represent predicted (hypothetical) mitotypes. Dashes on the lines connecting mitotypes indicate hypothesized mutational steps. Colored fields denote different localities where the species occur. *Note:* (*) The sequence of *S. rostrata* sp. nov. is based on a whole female specimen from Cantarranas that externally matches the description of *S. rostrata* sp. nov. This specimen was used for DNA extraction, leaving no material for further morphological confirmation.

The mitotype network was constructed using partial mtDNA CO1 sequences from *Stigmella lunacrescens* sp. nov., *S. mutabilis* sp. nov., *S. rostrata* sp. nov., and the related *S. foreroi* (Figure 25). In this network, the detected mitotypes were distinctly separated into four groups, corresponding to the morphological identification of the specimens. These groups were distinguished by at least 28 hypothesized mutational steps (between *S. mutabilis* sp. nov. PQ630806 and *S. foreroi*), while the largest difference within any single species was nine mutational steps (in *S. mutabilis* sp. nov.). This pattern suggests the presence of a barcoding gap between the analyzed species.

**Figure 26:**
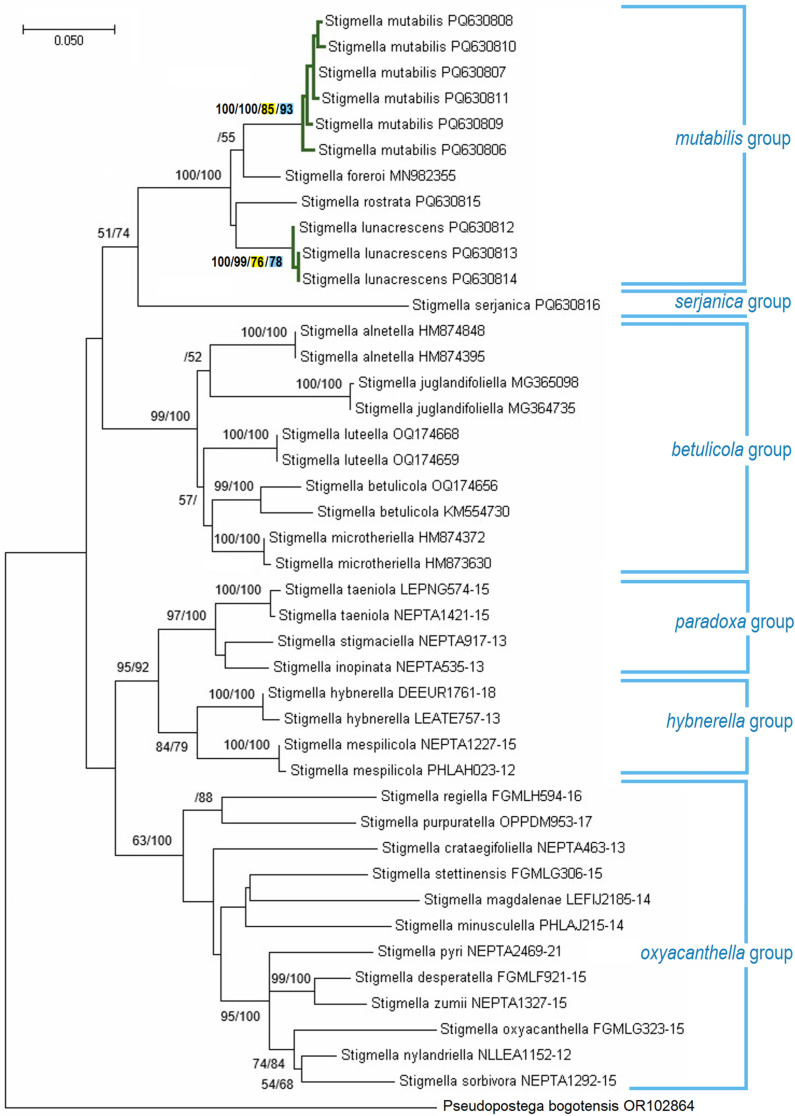
Phylogenetic relationships of *Stigmella* Schrank based on 657 bp-long mtDNA CO1-5′ sequences and the GTR + G + I evolutionary model; topology reconstructed using Maximum Likelihood and Bayesian inference. Branch statistics, separated by slashes: Maximum Likelihood probability (10,000 replicates)/Bayesian posterior probability (10,000,000 generations)/ASAP probability (yellow background)/bPTP support value (turquoise background) in % (only values above 50 are shown). Green clusters of the tree were supported as separate species by both ASAP and bPTP delimitation algorithms. *Pseudopostega bogotensis* (Opostegidae) was included as the outgroup.

**Figure 2:**
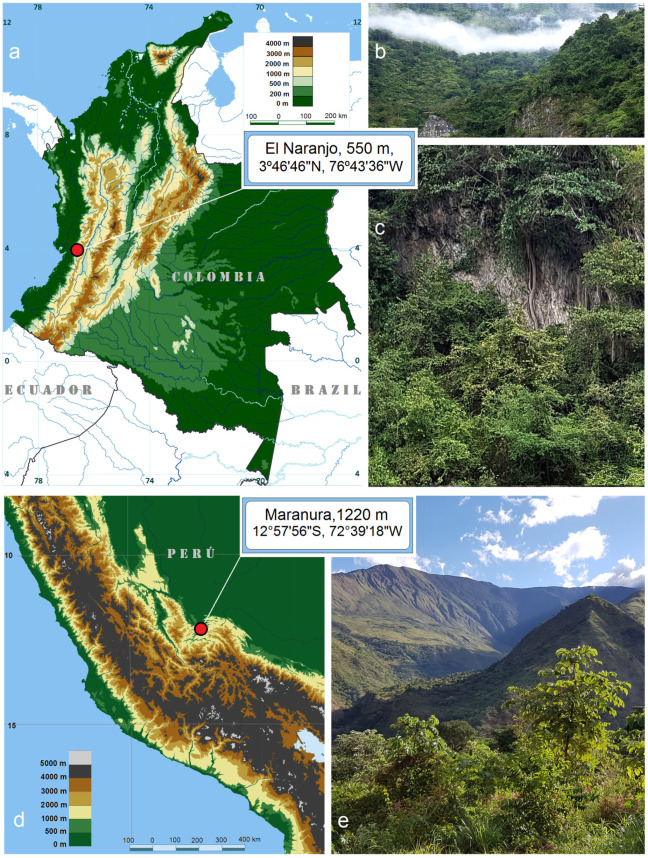
Study localities in Colombia and Peru from which additional, comparable material was collected for the present study: (**a**–**c**) El Naranjo, Dagua Municipality, Valle del Cauca Department, Colombia, with general views of the habitat; (**d**,**e**) Maranura, Cerro Quintalpata, La Convención Province, Peru, with general views of the habitat.

**Figure 6:**
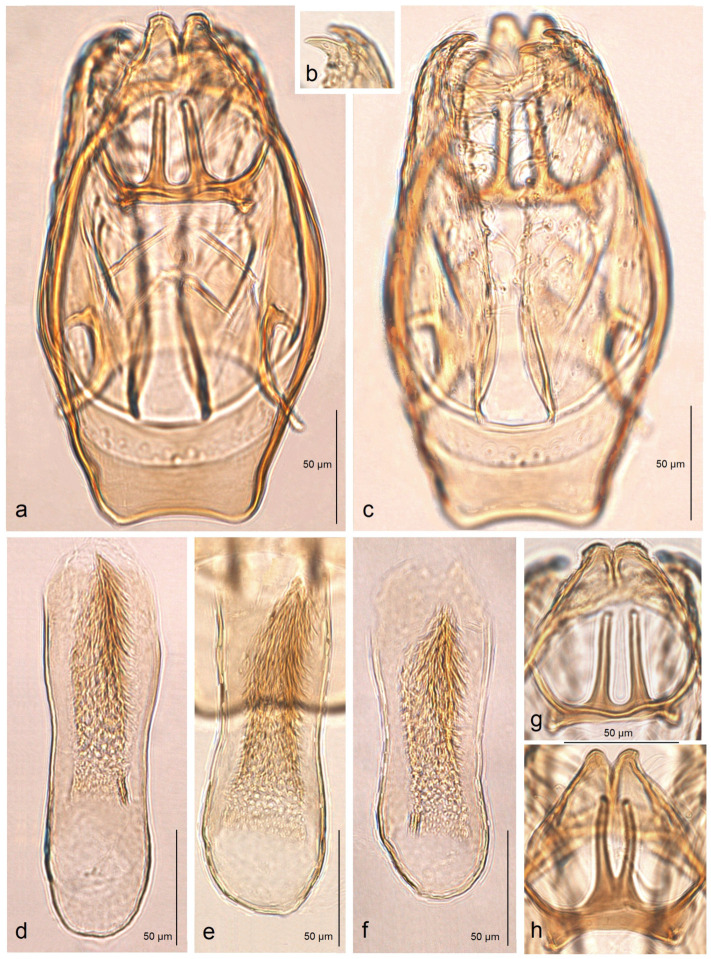
Male genitalia of *Stigmella ruralis* Stonis & Remeikis, sp. nov., from Cantarranas, the Río Choluteca Valley, Honduras: (**a**–**c**) genital capsule, holotype, genitalia slide RA1239 (MfN); (**d**) phallus, holotype, slide RA1239 (MfN); (**e**) the same, paratype, slide AD1199 (MfN); (**f**) the same, other paratype, slide RA1211 (MfN); (**g**) uncus and gnathos, paratype, slide RA1211 (MfN); (**h**) the same, paratype (aberrant), slide AD1199 (MfN).

**Figure 11:**
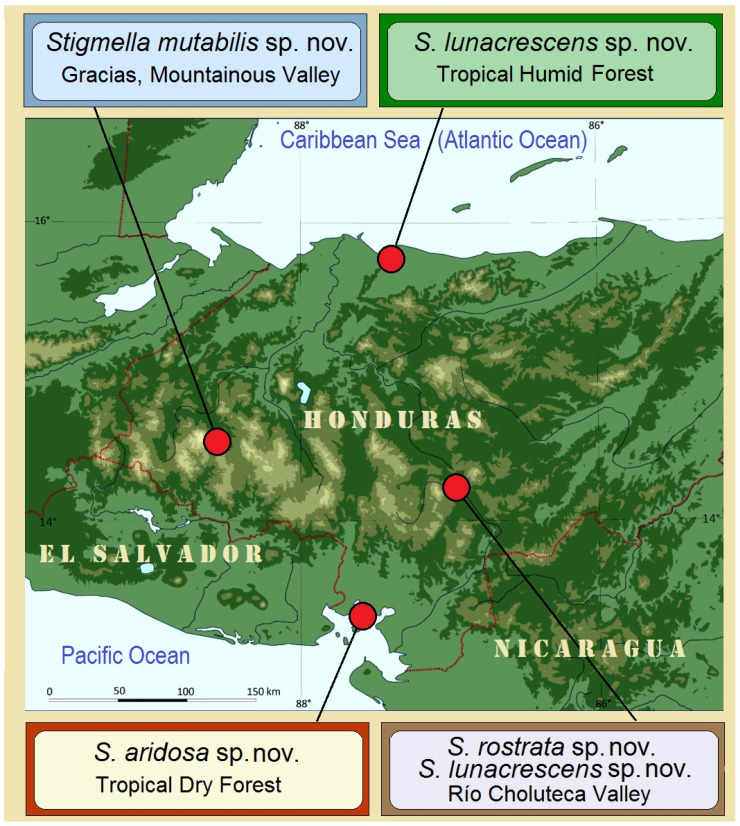
Discovery of *Stigmella mutabilis* sp. nov. and its affiliates from different study localities in Honduras.

**Figure 12:**
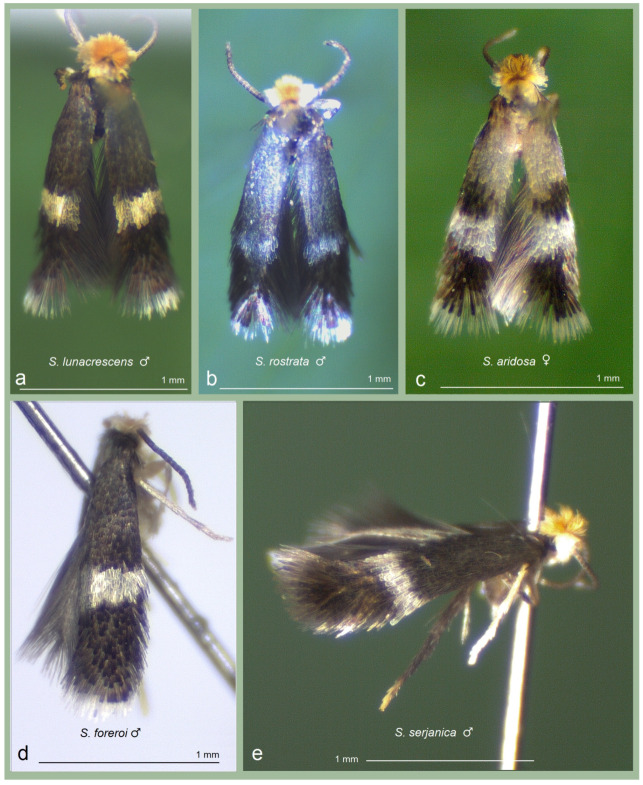
Adults of *Stigmella* species, morphologically and genetically related, documented from distinct, specifically selected study localities in Honduras, Colombia, and Peru: (**a**) *S. lunacrescens* Stonis & Remeikis, sp. nov., from Tela, the tropical humid forest on the Caribbean (Atlantic) coast of Honduras (MfN); (**b**) *S. rostrata* Stonis & Diškus, sp. nov., from Cantarranas, the Río Choluteca Valley, central mountainous Honduras (MfN); (**c**) *S. aridosa* Stonis & Remeikis, sp. nov., from the tropical dry forest on the Pacific coast of Honduras (MfN); (**d**) *S. foreroi* Stonis & Vargas, 2019, from El Naranjo, Departamento de Valle del Cauca, Municipio de Dagua, Colombia (MPUJ); (**e**) *S. serjanica* Stonis & Diškus, sp. nov., from Maranura, Cusco Region, La Convención Province, Cerro Quintalpata, Peru (MfN). *Note: Figure* (**c**) represents a female specimen.

**Figure 13:**
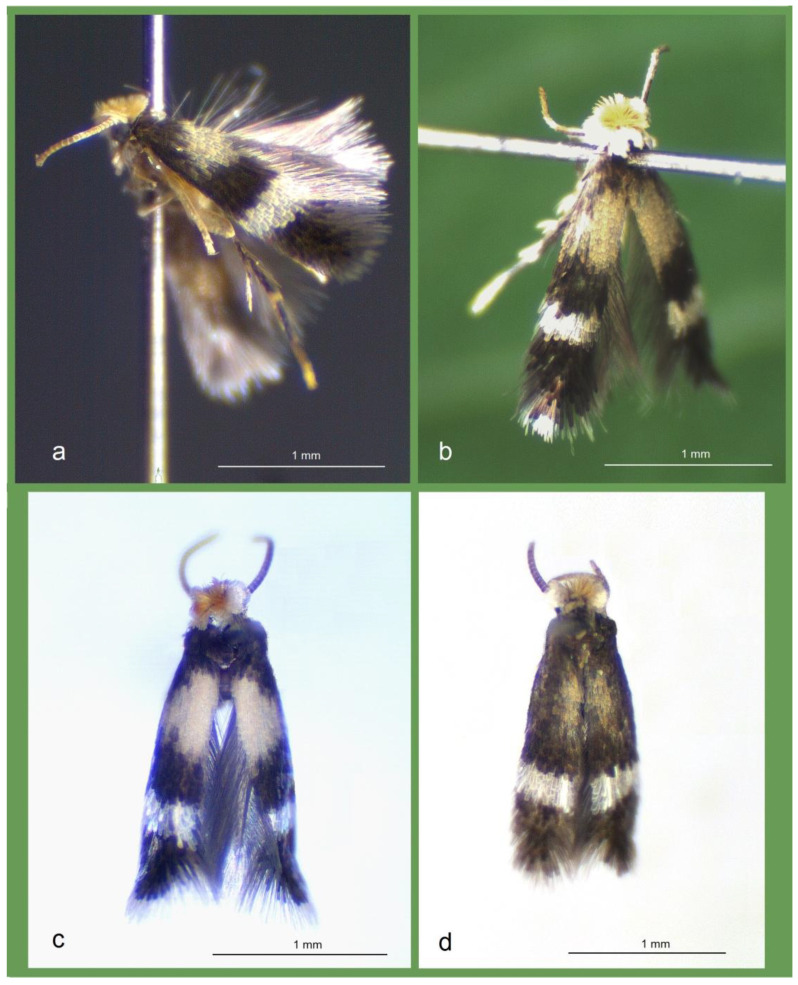
Adults of *Stigmella mutabilis* Stonis & Remeikis, sp. nov., discovered in Gracias, a mountainous valley in Lempira Department, Honduras: (**a**) the male holotype, with the genitalia slide label no. RA1193 (MfN); (**b**) the female paratype, with the genitalia slide label no. RA1200 (MfN); (**c**) the male paratype, with the genitalia slide label no. RA1195 (MfN); (**d**) the currently undissected male paratype, without a genitalia slide (MfN).

**Figure 21:**
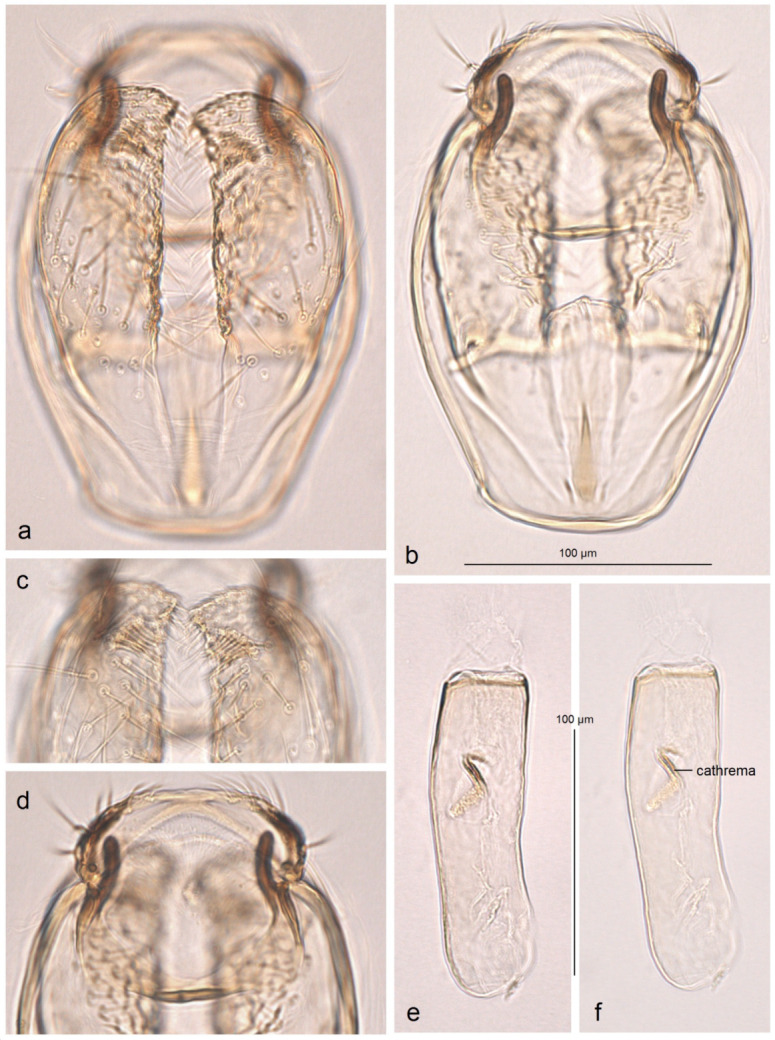
Male genitalia of *Stigmella aridosa* Stonis & Remeikis, sp. nov., holotype, from Pacific coast of Honduras, Isla del Tigre, Amapala, genitalia slide no. RA1231 (MfN): (**a**,**b**) genital capsule with phallus removed (mind the oval, rounded shape); (**c**) the same, valvae (note the dense cluster of setae); (**d**) the same, uncus and gnathos; (**e**,**f**) phallus.

**Figure 22:**
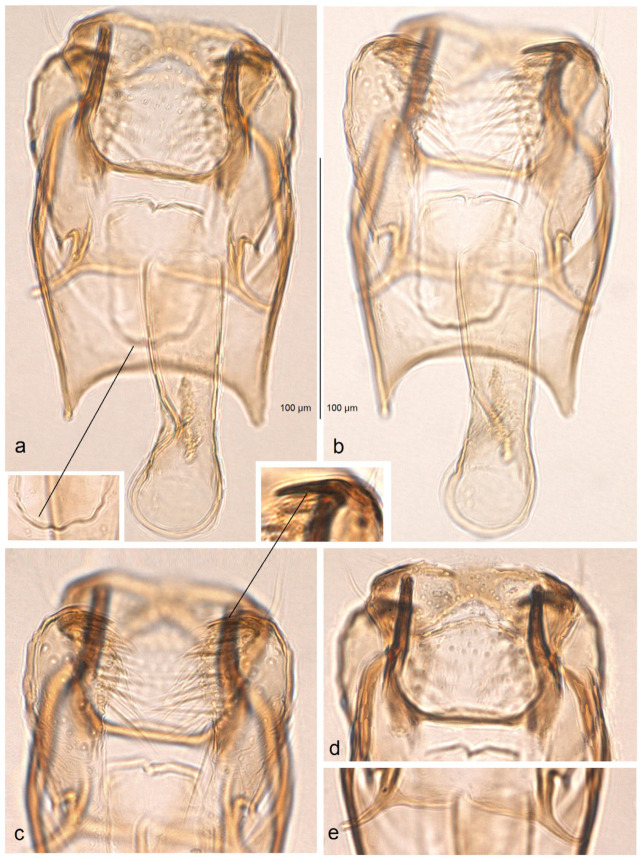
Male genitalia of *Stigmella rostrata* Stonis & Diškus, sp. nov., holotype, from Cantarranas, Morazán Department, ca. 660 m, genitalia slide no. AD1203 (MfN): (**a**,**b**) a view of genital capsule with phallus inside (note the wide juxta and the beak-like thickening of the valva; (**c**) valvae; (**d**) uncus and gnathos; (**e**) transtilla.

The ASAP and bPTP species delimitation algorithms, applied to species represented by multiple specimens, confirmed the genetic distinctness of *S. lunacrescens* sp. nov. (ASAP: 76%; bPTP: 78%) and *S. mutabilis* sp. nov. (ASAP: 85%; bPTP: 93%) (Figure 26).

As already mentioned, the analysis revealed unexpectedly high variability within *S. mutabilis* sp. nov., where mitotype diversity was at its maximum (Hd ± SD = 1.000 ± 0.096). All obtained sequences collected from the same location and time point were unique, differing by one (between PQ630807 and PQ630811) to nine (between PQ630806 and PQ630810) mutational steps (Figure 25). The abundance of hypothetical mitotypes suggests that the intraspecific diversity of *S. mutabilis* sp. nov. is significantly greater than the six mitotypes discovered so far.

#### 3.2.8. Designation of the *Stigmella mutabilis* and *S. serjanica* Species Groups

Species groups are not taxonomic units. In Nepticulidae, they are established primarily for diagnostic purposes, playing an important role in identifying (diagnosing) and providing orientation within highly speciose genera such as *Stigmella*. The main publications addressing species groups [9,14,29,43] have provided varying numbers of species groups globally, as well as for the Neotropical fauna of *Stigmella* (notably, [10]).

Although the four species analyzed in this study (*S. mutabilis* sp. nov., *S. lunacrescens* sp. nov., *S. aridosa* sp. nov., *S. rostrata* sp. nov.) from Honduras, along with *S. foreroi* from Colombia, are distinct taxa, they share a unique combination of morphological characters not observed in other *Stigmella* species. This shared combination makes them closely similar to one another, yet distinct from other species groups.

To support the hypothesis that these species form a cohesive group separate from other closely related species groups, we constructed multiple phylogenetic trees, which consistently yielded the same results. Based on partial mtDNA CO1 sequences of *Stigmella* species included in the mitotype network (Figure 25) and their morphologically closest relatives, the phylogenetic tree (Figure 26) revealed well-supported clades for all analyzed species groups: *S. betulicola*, *S. paradoxa*, *S. hybnerella*, and *S. oxyacanthella*.

The applied algorithms reliably separated *S. mutabilis* sp. nov., *S. foreroi*, *S. rostrata* sp. nov., and *S. lunacrescens* sp. nov. from other species, clustering them into a distinct branch with maximum support (ML bootstrap value and Bayesian posterior probability both equaled 100%). Additionally, these four species—plus *S. aridosa* sp. nov., which was not included in the molecular analysis due to the absence of available sequences—share several defining morphological characters: (1) dark forewings with a silvery shiny transverse fascia; (2) a rounded male genital capsule with a valva that is triangularly extended basally, though weakly chitinized; (3) a distinctive, unique, folded juxta; (4) an elaborated transtilla; (5) a lyra-shaped gnathos with caudal processes strongly chitinized and darkened caudally; (6) a large uncus with an X-shaped thickening; and (7) weakly developed or absent cornuti in the phallus. Furthermore, the host plants for all these species are presumed to belong to the genus *Serjania* Mill. (Sapindaceae). This assumption is based on one species being reared from *Serjania*, while three others were collected using light traps set near vacated leaf mines on this plant. Thus, based on both morphological and molecular evidence, *S. mutabilis* sp. nov., *S. lunacrescens* sp. nov., *S. aridosa* sp. nov., *S. rostrata* sp. nov., and *S. foreroi* are here designated as the members of the *S. mutabilis* group, a newly established species group.

The closest known species to this group (though not fully identical) is the Peruvian *S. serjanica* sp. nov., whose larvae also mine *Serjania*. However, despite this ecological similarity, its placement within the *S. mutabilis* group was not sufficiently supported by the Maximum Likelihood (ML bootstrap value: 51%) and Bayesian inference (Bayesian posterior probability: 74%) analyses, indicating that the phylogenetic position of this species remains unstable. Furthermore, regarding male genital morphology, *S. serjanica* sp. nov. lacks the gradually curved and basally extended valva characteristic of the *S. mutabilis* group. Instead, it possesses an apically bulged valva and a distinctive band of spine-like cornuti in the phallus, further distinguishing it from members of the *S. mutabilis* group.

Due to these morphological and molecular differences between *S. serjanica* sp. nov. and the *S. mutabilis* species group, we initially considered treating *S. serjanica* sp. nov. as a satellite species of the *S. mutabilis* group. However, we ultimately decided to establish *S. serjanica* sp. nov. as the sole member of a separate species group, the *S. serjanica* species group (designated here). We expect that more species of the *S. serjanica* group will be discovered in the future. Notably, the third author of this article (A.D.) observed intriguing vacated leaf mines on *Serjania* sp. during fieldwork in the Amazon Basin in 2025; however, no larvae or adults have been found so far.

## 4. Discussion

Pygmy moths (Nepticulidae), being speciose and often abundant across a wide range of terrestrial biomes, ecosystems, and habitats—from lowlands to high mountain zones (up to 4700 m elevation in the Andes; [10])—represent a highly suitable subject for a range of broader ecological and evolutionary studies [29].

This cosmopolitan lepidopteran family, which comprises the smallest moths in the world [10], belongs to one of the most basal phylogenetic lineages of the order Lepidoptera [44], with a geological origin estimated at approximately 130 million years ago [45]. This deep evolutionary history lends further theoretical significance to its study. In addition, the host plant specificity of their leaf-mining larvae—predominantly monophagous species—along with their presumed sensitivity to environmental conditions and limited dispersal potential, makes them particularly informative for ecological biogeography.

Honduras, although the second-largest country in Central America, is relatively small compared to major Neotropical biodiversity hotspots such as Colombia, Venezuela, Peru, and Brazil. With a land area of approximately 112,000 square kilometers, it stretches roughly 300 km from its Pacific coastline in the south to the Caribbean in the north. Despite its modest size, the country encompasses an impressive diversity of ecosystems—from tropical dry forests along the Pacific coast and in large interior valleys, to subtropical mountain zones and tropical lowland humid forests.

Based on our working hypothesis that a mosaic of habitats plays a key role in shaping the country’s biodiversity, our selection of Honduras as the study region proved highly productive. Moreover, as Honduras had previously remained almost entirely unexplored with respect to Nepticulidae diversity, every new record from the country is of particular scientific value.

Our study, conducted across carefully selected and ecologically distinct study plots, resulted in the discovery and formal description of several closely related but distinct species. In Case Study 1, we described *Stigmella ruralis* sp. nov. from the Río Choluteca valley, *S. pacifista* sp. nov. from the tropical dry forests along the Pacific coast of Honduras, and *S. atlantidica* sp. nov. from the tropical humid forests along the Caribbean coast. In Case Study 2, we described *S. mutabilis* sp. nov. from a mountainous valley in the Lempira Department, *S. lunacrescens* sp. nov. from the tropical humid forests of the Caribbean coast, *S. rostrata* sp. nov. from the Río Choluteca valley in the central mountainous region, and *S. aridosa* sp. nov. from the tropical dry forest along the Pacific coast. In addition, these Central American taxa were compared with morphologically similar species from South America—*S. foreroi* Stonis & Vargas from Colombia and *S. serjanica* sp. nov. from Peru. In total, the present study resulted in the description of eight closely related yet genetically and morphologically distinct new species.

The distinctiveness of these closely related species was supported not only by morphological investigations but also by molecular sequence analysis. Species delimitation using the ASAP and bPTP algorithms, applied to taxa represented by multiple specimens, confirmed their genetic distinctness. For instance, *S. lunacrescens* sp. nov. was supported as a separate species with high confidence (ASAP: 76%; bPTP: 78%), as was *S. mutabilis* sp. nov. (ASAP: 85%; bPTP: 93%). Notably, *S. mutabilis* sp. nov. exhibited unexpectedly high intraspecific variability, with haplotype diversity reaching its maximum (Hd ± SD = 1.000 ± 0.096).

One of the most striking findings of this study is the delimitation between *S. ruralis* sp. nov. and the closely related species *S. pacifista* sp. nov. At first glance, these two species appear so similar that, at the beginning of our study, we considered them to be a single species. However, a detailed examination of the male genitalia suggested that they are distinct. Ultimately, molecular analyses provided even stronger evidence supporting this distinction.

In our mitotype network (Figure 8c), we observe that there are 23 mutational steps between the predicted (hypothetical) mitotype and *S. pacifista* sp. nov., 28 steps between the hypothetical mitotype and *S. ruralis* sp. nov., and 32 steps between the hypothetical mitotype and the Peruvian *S. molinensis*. This also means that there are 55 mutational steps between *S. pacifista* sp. nov. and the phylogenetically much more distant *S. molinensis*, while there are still 51 steps between the two Honduran species, *S. pacifista* sp. nov. and *S. ruralis* sp. nov.

It is worth noting that *S. pacifista* sp. nov. (from Isla Zacate Grande on the Pacific coast of Honduras) and *S. ruralis* sp. nov. (from Cantarranas, in the Río Choluteca valley) were collected from localities only about 100 km apart (in a straight line), yet these locations differ significantly in environmental conditions and, consequently, in host plants.

Given that the primary goal of our study was to provide evidence—based on a little-studied lepidopteran family, Nepticulidae—that closely situated but environmentally distinct localities, and therefore likely to support different host plants, can harbor different species, our findings offer strong evidence.

The discovery of several closely related yet distinct Nepticulidae taxa in separate habitats reinforces our hypothesis that environmental heterogeneity acts as a “hidden engine” driving and amplifying tropical biodiversity. We propose that environmental differences among localities exert selective pressures that encourage species to adapt to specific ecological niches. As a result, we now observe a fascinating phenomenon: species that likely share a common ancestor have undergone divergence over time. This leads to taxa that are morphologically and genetically similar in many respects, yet clearly distinct—an outcome that strongly exemplifies the process of adaptive radiation.

In this context, it is noteworthy that our parallel study in Honduras yielded analogous results. At first glance, the minute, dark-colored moths—characterized by a shiny fascia on the forewing, distally darkened antennae, particularly long and slender lateral lobes of the vinculum (a distinctive trait in male genitalia), a partially reduced phallus tube, and slightly dentate cornuti on the vesica—appeared to belong to a single species. However, a more detailed examination of both male and female genital structures, and most importantly, molecular analysis, revealed the presence of two closely related but distinct species: *Stigmella minicontorta* Stonis & Remeikis and *S. gracifurcata* Stonis & Remeikis [12]. Interestingly, *S. gracifurcata* occurs along the Caribbean coast in the tropical humid forests, while *S. minicontorta* inhabits the tropical dry forests along the Pacific coast of Honduras. Though their collection localities are separated by only about 260 km, the climatic and biotic differences between these two forest ecosystems are striking. These findings support the interpretation that *S. minicontorta* and *S. gracifurcata*—two species likely derived from a shared ancestor—represent a case of ecological vicariance—driven not by geographic distance or physical barriers, but by contrasting environmental conditions and, presumably, divergent (yet to be identified) host plant associations.

In this context, it is also worth noting that during our study in Honduras, we encountered a similar case: three distinct trumpet moth species, all belonging to the small endemic genus *Coptotrichoides* Diškus & Stonis (Tischeriidae), were found in four tropical localities that are geographically close yet differ markedly in their climatic and biotic conditions (unpublished). Nearly every locality yielded a distinct species, except for Cantarranas (in the Río Choluteca valley), where the same species also occurs in Tela (the tropical humid forests along the Caribbean coast).

The remarkable diversity of Nepticulidae documented in Honduras provides compelling evidence of the influence of ecological heterogeneity and evolutionary processes. Honduras thus offers an exemplary model for understanding how a mosaic of distinct ecosystems can amplify biodiversity. Understanding this multiplicative effect is important not only for deepening our knowledge of evolutionary dynamics and species adaptation but also for informing and advancing strategies in biodiversity conservation.

## Data Availability

The molecular data presented in this study can be found in an online repository (GenBank). Open access depositories of the collection material (physical specimens and their genitalia mounts, including all described new species) are listed in the article.
